# Hedgehog-interacting protein orchestrates alveologenesis and protects against bronchopulmonary dysplasia and emphysema

**DOI:** 10.1126/sciadv.adu2958

**Published:** 2025-05-07

**Authors:** Datian Ye, Shiyun Feng, Xinguo Yang, Yanjing Su, Jing Zhang, Haixin Feng, Minqi Zhou, Bin Zhou, Lihui Duan, Tien Peng, Chaoqun Wang

**Affiliations:** ^1^ Zhongshan Institute for Drug Discovery, Shanghai Institute of Materia Medica, Chinese Academy of Sciences, Zhongshan 528400, China.; ^2^ Perfect Life Science Research Institute, Perfect (GuangDong) Co. Ltd., Zhongshan 528402, China.; ^3^ School of Pharmaceutical Sciences, Southern Medical University, Guangzhou 510515, China.; ^4^ School of Chinese Materia Medica, Nanjing University of Chinese Medicine, Nanjing 210023, China.; ^5^ State Key Laboratory of Molecular Development Biology, Institute of Genetics and Developmental Biology, Chinese Academy of Sciences, Beijing 100101, China.; ^6^ Department of Medicine, University of California, San Francisco, San Francisco, CA 94143, USA.; ^7^ University of Chinese Academy of Sciences, Beijing 100049, China.; ^8^ New Cornerstone Science Laboratory, Key Laboratory of Multi-Cell Systems, Shanghai Institute of Biochemistry and Cell Biology, Center for Excellence in Molecular Cell Science, Chinese Academy of Sciences, Shanghai 200031, China.; ^9^ State Key Laboratory of Drug Research, Shanghai Institute of Materia Medica, Chinese Academy of Sciences, Shanghai 201203, China.

## Abstract

Most of the lung’s gas-exchange surface forms during alveologenesis and its disruption causes bronchopulmonary dysplasia (BPD) in infants, characterized by alveolar simplification and myofibroblast accumulation. BPD also increases the risk of adult emphysema, marked by alveolar loss. Despite this connection, mechanisms linking these conditions and effective treatments are still lacking. We identify hedgehog-interacting protein (*HHIP*), associated with both BPD and emphysema, as a critical regulator of alveologenesis. During this process, *Hhip*-expressing cells expanded, accompanied by hedgehog (Hh) signaling inhibition and myofibroblast transition. Stromal-specific *Hhip* deletion led to hyperactivation of Hh-IGF1 signaling axis, causing persistent SMA^+^ myofibroblasts and epithelial stem/progenitor cell senescence. Hyperactivation of this pathway was also observed in human BPD and hyperoxia-induced BPD models. Early *Hhip* deficiency resulted in adult emphysema with myofibroblast accumulation. We developed a therapeutic Fc-fused HHIP protein that mitigated BPD in neonatal mice and prevented adult emphysema. These findings establish HHIP as a critical regulator of alveologenesis and a therapeutic target for BPD and emphysema.

## Teaser

Hedgehog-interacting protein plays a pivotal role in lung maturation and resistance to chronic diseases.

## INTRODUCTION

Alveologenesis is the final and vital phase of lung development after the saccular stage. It initiates on postnatal day (P) 3 and continues until P39 in mice (*1*–*3*). The process is further subdivided into two phases: classical alveologenesis (P3-P14) and continued alveologenesis (P15-P39). During the classical phase, the division of alveolar sacs by the formation of secondary septa leads to a drastic expansion of gas-exchange surface. In the continued phase, the increase of alveolar surface is largely attributed to simple volume expansion concomitant with the growth of the lung and the chest cavity. Myofibroblasts in the alveoli, characterized by the coexpression of α–smooth muscle actin (SMA) and platelet-derived growth factor receptor–α (PDGFRα), play a critical role in the formation of secondary septa during classical alveologenesis. These cells typically regress as lung development progresses beyond this phase (*4*–*7*). The dysregulation of myofibroblast formation and disappearance during alveologenesis has been consistently associated with alveolar malformations (*6*, *8*–*10*). However, the precise mechanisms underlying this process remain poorly understood.

Bronchopulmonary dysplasia (BPD), characterized by alveolar simplification, is the most common and serious chronic lung disease in preterm infants (*11*). The unclear mechanisms of BPD and incomplete understanding of alveologenesis hinder the development of effective treatments. Lung tissues of patients with BPD demonstrate aberrant persistence of myofibroblasts, highlighting their role as a clinically relevant feature of BPD and a potential therapeutic target (*12*).

Chronic obstructive pulmonary disease (COPD) is the third leading cause of death worldwide. As a major type of COPD, emphysema is characterized by alveolar loss, resulting in enlarged airspaces and a reduced surface area for gas exchange (*13*). This condition shares a similar alveolar phenotype with BPD (*14*). Notably, individuals with childhood BPD history have a higher risk of developing emphysema in adulthood (*15*, *16*). Thus, understanding the mechanisms of alveologenesis and BPD pathogenesis could reveal therapeutic targets for both conditions.

Hedgehog (Hh)–interacting protein (*HHIP*) is a disease-susceptibility gene for both BPD and COPD (*17*–*20*). HHIP acts as a negative regulator of Hh signaling by sequestering SHH, thereby modulating lung stromal-epithelial interactions (*21*, *22*). We have previously showed that *Hhip* deletion and Hh activation disrupt stromal cell function in adult lung (*23*, *24*). In this study, we set out to define HHIP’s role in regulating alveologenesis and BPD pathogenesis. Our findings demonstrate that HHIP drives myofibroblast transition, inhibits epithelial stem/progenitor cell senescence, and guards against BPD and BPD-associated emphysema via suppressing Hh-IGF1 signaling axis.

## RESULTS

### Alveolar expansion of *Hhip*-expressing cells is accompanied by Hh inhibition and myofibroblast transition

**Fig. 1.**
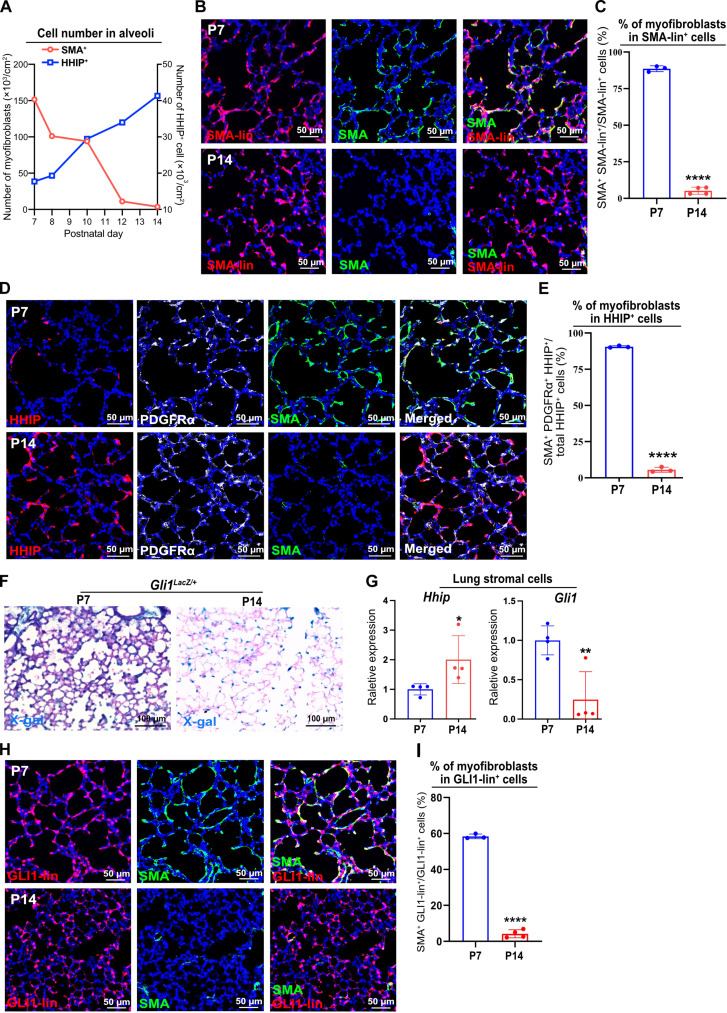
Expansion of HHIP^+^ cells, Hh inhibition, and myofibroblast transition during alveologenesis. (**A**) Quantification of SMA^+^ and HHIP^+^ cells in the alveoli during alveologenesis. (**B**) Immunofluorescence (IF) analysis of SMA-lineage cells (staining tdT) and SMA in the alveoli of *Acta2^DreER/+^:R26R^RSR-tdT/+^* mice at P7 and P14. (**C**) Histology quantification of the percentage of SMA^+^ myofibroblasts in total SMA-lineage cells. (**D**) IF analysis of HHIP, PDGFRα, and SMA expression in the alveoli. (**E**) Histology quantification of the percentage of myofibroblasts in HHIP^+^ cells. (**F**) X-gal staining of *Gli1^lacZ/+^* reporter demonstrates *Gli1* expression in the alveoli of P7 and P14 mice. (**G**) qPCR analysis of *Hhip* and *Gli1* expression in the lung stromal cells of P7 and P14 mice. (**H**) IF analysis of GLI1-lineage cells (staining tdT) and SMA in the alveoli of *Gli1^CreER/+^:R26R^tdT/+^*mice. (**I**) Histology quantification of the percentage of myofibroblasts in total GLI1-lineage cells. Each data point represents one mouse [(A), (C), (E), (G), and (I)] of an individual experiment. Data are expressed as mean ± SD. *P < 0.05, **P < 0.005, and ****P < 0.0001.

Alveologenesis is a complex process involving substantial changes in cell type composition, in particular the dynamics of myofibroblasts in the alveoli. We set out to examine the temporal changes of myofibroblasts by staining SMA during this process and revealed a timely decline: Myofibroblast population exhibited a gradual decrease from P7 to P14 (Fig. 1A and fig. S1A), as previously reported (*5*). To further explore whether myofibroblasts died or became SMA negative (SMA^−^) after P7, we generated *Acta2^DreER/+^:R26R^RSR-tdT/+^* mice, allowing us to trace SMA-lineage cells. SMA-lineage cells were widely present in the alveoli at both P7 and P14 (Fig. 1B). Costaining with SMA showed that 95% of myofibroblasts in the alveoli were effectively labeled in our SMA-lineage tracing animals (Fig. 1B and fig. S1B). Among these SMA-lineage cells, approximately 90% were SMA positive (SMA^+^) at P7. However, by P14, the majority had transitioned to SMA^−^ (Fig. 1C). These findings suggest that, between P7 and P14, myofibroblasts undergo a phenotypic change from SMA^+^ to SMA^−^, rather than being lost. We refer to this process as myofibroblast transition.

Notably, we observed a concurrent rise in the number of *Hhip*-expressing cells in the alveoli as SMA^+^ myofibroblasts decreased from P7 to P14 (Fig. 1A and fig. S1A). Colocalization of HHIP with SMA and PDGFRα showed that the majority of HHIP^+^ cells were positive for these markers at P7, indicating that these cells were myofibroblasts in the alveoli (Fig. 1, D and E). However, by P14, the majority of HHIP^+^ cells turned SMA^−^ (Fig. 1, D and E). Myofibroblasts in the alveoli consist of two populations: CDH4^−^ alveolar myofibroblasts (ALMFs) surrounding alveoli and CDH4^+^ ductal myofibroblasts (DMFs) surrounding alveolar ducts (*25*). Immunostaining analysis showed that *Hhip* was expressed in both ALMFs and DMFs at P7 (fig. S1C).

As a negative regulator of Hh signaling, HHIP suppresses Hh activation by sequestering sonic hedgehog ligand (SHH). Consistent with the increased expression of *Hhip*, we found that the number of cells expressing *Gli1*, a transcriptional readout of Hh activation (*26*), substantially decreased in the alveoli by P14 (Fig. 1F), as previously reported (*27*). Quantitative polymerase chain reaction (qPCR) analysis of lung stromal cells confirmed the above findings, demonstrating up-regulation of *Hhip* and down-regulation of *Gli1* expression during alveologenesis (Fig. 1G). Our previous study demonstrated that Gli1^+^ cells are largely absent in the alveoli of adult lungs, while Gli2^+^ cells are abundantly present (*23*). During alveologenesis, RNA in situ hybridization for *Gli2* and *Gli1* expression, combined with PDGFRα immunostaining, revealed that Gli2^+^ cells were widely present in alveolar stromal cells at both P7 and P14, with all Gli1^+^ cells also expressing *Gli2* (fig. S1, D to F). Consistent with the *Gli1^LacZ/+^* reporter, *Gli1* expression decreased significantly at P14 compared with P7 (fig. S1, D and G). In addition, *Gli2* RNA in situ analysis of SMA-lineage cells at P14 revealed that most of these cells were Gli2^+^, with minimal *Gli1* expression (fig. S1, H and I). These data suggest that Gli1-expressing cells constitute a subset of Gli2^+^ cells. The sustained expression of *Gli2* in these cells appears to be independent of active Hh signaling, in line with our earlier observations in adult lungs (*23*).

To further explore whether alveolar *Gli1*-expressing cells decreased in number due to Hh signaling inhibition or cell death, we labeled GLI1-lineage cells at birth. Lineage-tracing analysis demonstrated that GLI1-lineage cells widely presented in the alveoli at both P7 and P14 (Fig. 1H), indicating that the decrease in the number of *Gli1*-expressing cells was caused by Hh inhibition. Consistent with the findings from SMA-lineage tracing, around 60% of GLI1-lineage cells were SMA^+^ at P7, when Hh was active. However, by P14, when Hh was inhibited, less than 5% of these GLI1-lineage cells remained SMA^+^ (Fig. 1, H and I). Of note, approximately 90% of myofibroblast population was marked by GLI1-lineage cells at both P7 and P14 (fig. S1J). Immunostaining analysis of CDH4 and HHIP in SMA-lineage and GLI1-lineage cells revealed a significant increase in the ratio of DMFs from P7 to P14 (fig. S2, A to D). Together, these data indicate a potential link between myofibroblast transition and Hh signaling inhibition, suggesting that HHIP likely modulate both Hh activation and myofibroblast transition.

### HHIP down-regulates the stromal Hh-IGF1 signaling to drive myofibroblast transition

**Fig. 2.**
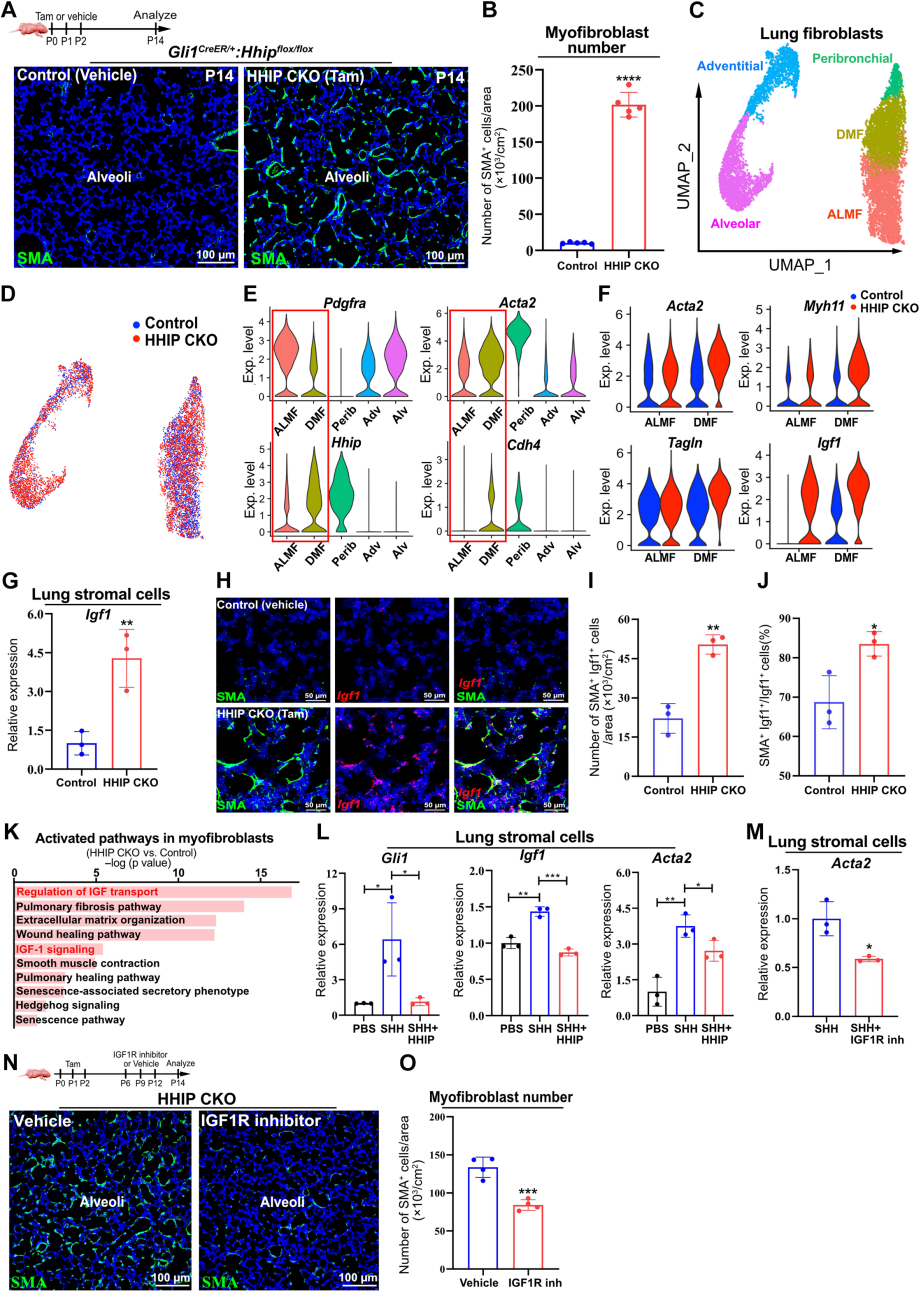
*Hhip* deletion induces the persistence of myofibroblasts. (**A**) IF analysis of SMA in the alveoli of *Hhip*-deleted (HHIP CKO) and control mice at P14. (**B**) Number of myofibroblasts per unit alveolar area of *Hhip*-deleted and control mice. (**C** and **D**) UMAP showing cell clusters in the lung fibroblasts of *Hhip*-deleted and control mice at P14. (**E**) Violin plots showing the expression of *Pdgfra*, *Acta2*, *Hhip*, and *Cdh4* in alveolar myofibroblasts (ALMF), ductal myofibroblasts (DMF), peribronchial fibroblasts (Perib), adventitial fibroblasts (Adv), and alveolar fibroblasts (Alv). (**F**) Expression of *Acta2*, *Myh11*, *Tagln*, and *Igf1* in ALMFs and DMFs of *Hhip*-deleted and control mice. (**G**) qPCR analysis of *Igf1* expression in the lung stromal cells isolated from *Hhip*-deleted and control mice. (**H**) Analysis of *Igf1* (RNA in situ*)* and SMA expression in the alveoli. (**I**) Number of SMA^+^ Igf1^+^ cells per unit alveolar area of *Hhip*-deleted and control mice at P14. (**J**) Percentage of SMA^+^ Igf1^+^ cells in total Igf1^+^ cells. (**K**) Top 10 activated pathways in *Hhip*-deleted, relative to control myofibroblasts, analyzed with IPA. (**L**) qPCR analysis of *Gli1*, *Igf1*, and *Acta2* expression in the lung stromal cells treated with PBS, SHH, and SHH plus HHIP. (**M**) qPCR analysis of *Acta2* expression in SHH-stimulated lung stromal cells treated with vehicle or IGF1R inhibitor. (**N**) IF analysis of SMA expression in the alveoli of *Hhip*-deleted mice administered with vehicle or IGF1R inhibitor. (**O**) Number of myofibroblasts per unit alveolar area of *Hhip*-deleted mice administered with vehicle or IGF1R inhibitor. All in vitro experiments have been repeated at least one time with consistent results for validation. Each data point represents one mouse [(B), (G), (I), (J), and (O)] of an individual experiment. Data are expressed as mean ± SD. *P < 0.05, **P < 0.005, ***P < 0.0005, and ****P < 0.0001.

To determine whether and how HHIP modulates myofibroblast transition during neonatal alveolarization, we generated *Hhip* conditional knockout mice *Gli1^CreER/+^:Hhip^flox/flox^* (hereafter referred to as *Gli1^HHIPCKO^*). Deletion of *Hhip* resulted in a significant increase in the number of SMA^+^ myofibroblasts at P14, compared with control lungs (Fig. 2, A and B). Consistently, flow cytometry analysis revealed a significant increase in the percentage of SMA^+^ myofibroblasts among the PDGFRα^+^ stromal cells, as well as within total lung stromal cells, in P14 *Gli1^HHIPCKO^* mice compared with controls (fig. S3, A to C). Colocalization of SMA with CDH4 and PDGFRα revealed that the loss of *Hhip* led to an increased number of both SMA^+^ ALMFs and SMA^+^ DMFs (fig. S3, D to F). qPCR analysis of the lung stromal cells isolated from *Gli1^HHIPCKO^* confirmed loss of *Hhip* and up-regulation of *Acta2* (encoding SMA), along with induction of *Gli1*, indicating Hh activation after *Hhip* deletion (fig. S3G). To further assess the effect of Hh activation on myofibroblasts, we used a mouse model, *Gli1^CreER/+^:R26R^SmoM2/tdT^* (hereafter referred to as *Gli1^SMO^*), wherein constitutively active SmoM2 (*28*) could be induced to persistently activate Hh signaling in GLI1-lineage cells. In line with the findings from *Gli1^HHIPCKO^* mice, histological analysis of *Gli1^SMO^* lungs at P14 revealed a significant rise in SMA^+^ myofibroblast abundance (fig. S3, H to J). These data suggest that HHIP regulates the transition of myofibroblasts by restricting Hh activation.

To further characterize the functional role of HHIP in regulating myofibroblast transition, we performed single-cell RNA sequencing (scRNA-seq) on lung fibroblasts of *Gli1^HHIPCKO^* and the control mice at P14. Clustering analysis identified five distinct fibroblast clusters in the P14 lung (Fig. 2, C and D). Signature gene analysis for each subset (Fig. 2E, fig. S3K, and table S1) characterized them as ALMFs (Pdgfra^hi^/Acta2^+^/Hhip^+^), DMFs (Pdgfra^+^/Acta2^+^/Hhip^hi^/CDH4^+^), peribronchial fibroblasts (Pdgfra^−^/Acta2^+^/Cd9^hi^), adventitial fibroblasts (Pdgfra^+^/Dcn^hi^/Pi16^hi^), and alveolar fibroblasts (Pdgfra^+^/Npnt^hi^/Scube2^hi^), as we and others previously reported (*23*, *25*, *29*). Comparison of signature genes between ALMFs and DMFs revealed a high degree of concordance in marker gene expression (fig. S3L). Gene ontology (GO) analysis of their signature genes showed that both ALMFs and DMFs were enriched in GO terms related to extracellular matrix organization, branching morphogenesis, and cell growth (fig. S3, M and N). Notable differences included ALMFs being enriched in the regulation of kinase activity, while DMFs were enriched in contractile fiber. Differentially expressed gene (DEG) analysis of myofibroblasts between *Gli1^HHIPCKO^* versus control confirmed altered myofibroblast transition in *Gli1^HHIPCKO^*, indicated by significant up-regulation of myofibroblast signature genes (*Acta2*, *Myh11*, and *Tagln*) in both ALMFs and DMFs (Fig. 2F and tables S2 and S3). *Acta2* is also expressed in smooth muscle cells and pericytes. We further performed scRNA-seq analysis on these populations from *Gli1^HHIPCKO^* and control lungs, revealing no significant up-regulation of *Acta2* expression in either population following *Hhip* deletion (fig. S4, A to D).

Notably, *Igf1* exhibits the most pronounced up-regulation among the DEGs of myofibroblasts between *Gli1^HHIPCKO^* versus control (Fig. 2F and tables S2 and S3), as further confirmed by qPCR and RNA in situ analysis (Fig. 2, G to J), suggesting activation of IGF1 signaling in *Gli1^HHIPCKO^*. To further dissect molecular mechanisms underlying myofibroblast alteration, we performed ingenuity pathway analysis (IPA) analysis of the DEGs (*Gli1^HHIPCKO^* versus control). Consistently, IGF1 signaling was predicted as one of the top activated pathways in *Gli1^HHIPCKO^* myofibroblasts (Fig. 2K). To gain a more comprehensive understanding of the expression patterns of *Igf1* and its receptor *Igf1r*, we re-analyzed publicly available scRNA-seq data from Chen’s group (*25*), which includes all lung cell types. The analysis revealed that *Igf1* is predominantly expressed in stromal cells, including ALMFs and DMFs (fig. S4E). *Igf1r* exhibits a broader expression profile, being detected in stromal cells such as ALMFs and DMFs, as well as in alveolar and airway epithelium, endothelium, and immune cells (fig. S4F). In our stromal cell analysis, besides ALMFs and DMFs, we also observed an up-regulation of *Igf1* in peribronchial fibroblasts, with a modest increase in adventitial fibroblasts of *Gli1^HHIPCKO^* (fig. S4G). No significant increase in *Igf1* expression was detected in alveolar fibroblasts. These findings suggest that IGF1 may play a critical role in HHIP-modulated myofibroblast transition during alveologenesis.

Next, we defined the signaling cascade that caused the aberrant myofibroblast transition in vitro. Accumulating evidence has shown that *Acta2* expression increases in primary stromal cells after culture (*30*). Our qPCR analysis revealed that lung stromal cells from adult mice exhibited a continuous increase in *Acta2* expression with each passage (fig. S4H). In contrast, lung mesenchymal cells from neonatal mice (P12) did not show significant up-regulation of *Acta2* up to passage 4, with a noticeable increase occurring at passage 5 (fig. S4I). To minimize the impact of in vitro culture on *Acta2* elevation, we used lung stromal cells from P12 neonatal mice and restricted the passage number to below 5 in the following experiments. qPCR analysis of the lung stromal cells originally isolated from neonatal mice (P12) demonstrated that addition of recombinant SHH activated the expression of *Gli1*, *Igf1*, and *Acta2* in cultured lung stromal cells, and addition of recombinant HHIP abrogated Hh activation and the induction of *Igf1* and *Acta2* (Fig. 2L). Of note, addition of insulin-like growth factor 1 (IGF1R) inhibitor to SHH-stimulated stromal cells suppressed *Acta2* induction (Fig. 2M), suggesting that SHH promotes *Acta2* expression via IGF1. qPCR analysis of lung stromal cells treated with recombinant IGF1 demonstrated a significant up-regulation of *Acta2* expression (fig. S4J). Our previous study demonstrated that Hh activation in adult lung stromal cells suppresses the expression of secreted factors *Hgf* and *Wnt2* (*23*). Here, qPCR analysis of lung stromal cells from neonatal *Gli1^HHIPCKO^* mice revealed no significant differences in *Hgf* and *Wnt2* expression, although there was a trend toward decreased expression of both factors (fig. S4K). These findings demonstrate that HHIP drives myofibroblast transition primarily through an Hh-IGF1 signaling axis in a cell-autonomous manner.

To test whether IGF1 signaling blockade can rescue myofibroblast transition in vivo, we administered IGF1R inhibitor into our *Gli1^HHIPCKO^* postnatal animals. Immunofluorescence staining of the P14 lungs revealed a significant reduction of myofibroblasts within the alveoli of the IGF1R inhibitor–treated animals (Fig. 2, N and O). Together, these findings suggest that HHIP suppresses Hh-IGF1 signaling to drive myofibroblast transition during alveologenesis.

### HHIP deletion causes cell senescence via stromal IGF1

**Fig. 3.**
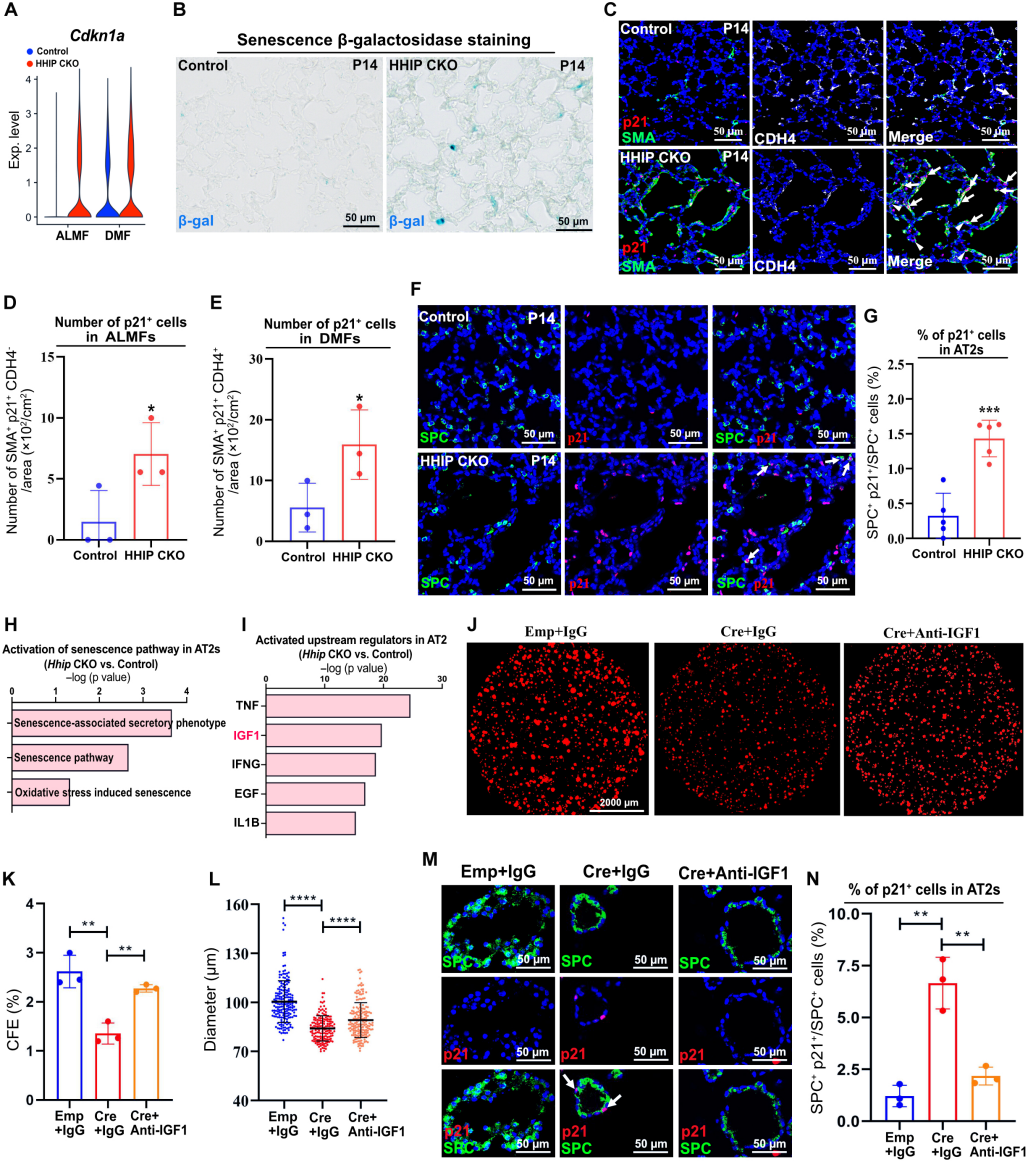
Deletion of *Hhip* induces cell senescence. (**A**) Violin plots showing the expression of *Cdkn1a* in ALMFs and DMFs of *Hhip*-deleted and control mice. (**B**) Senescence β-galactosidase staining of *Hhip*-deleted and control lungs. (**C**) IF analysis of SMA, p21, and CDH4 in *Hhip*-deleted and control lungs. Arrow: p21^+^ DMFs; arrowhead: p21^+^ ALMFs. (**D** and **E**) Number of p21^+^ ALMFs (D) and DMFs (E) per unit alveolar area of *Hhip*-deleted and control mice at P14. (**F**) IF analysis of SPC and p21 in *Hhip*-deleted and control lungs. Arrow: p21^+^ SPC^+^ cells. (**G**) Percentage of p21^+^ cells in AT2s. (**H**) Activation of senescence pathways in the AT2s of *Hhip*-deleted mice, relative to control AT2s, and analyzed with IPA. (**I**) Top 5 upstream regulators in the AT2s of *Hhip*-deleted mice, relative to control AT2s, analyzed with IPA. (**J**) AT2 organoids cocultured with lung stromal cells (*R26R^SmoM2/+^*) pre-infected with adenovirus-empty and adenovirus-Cre, treated with anti-IGF1 antibody and IgG. (**K** and **L**) Quantification of colony-forming efficiency (CFE) and organoid size. (**M**) IF analysis of SPC and p21 in AT2 organoids. Arrow: p21^+^ SPC^+^ cells. (**N**) Percentage of p21^+^ cells in AT2s in the organoid assay. All in vitro experiments have been repeated at least one time with consistent results for validation. Each data point represents one mouse [(D), (E), and (G)] of an individual experiment. Data are expressed as Mean ± SD. *P < 0.05, **P < 0.005, ***P < 0.0005, and ****P < 0.0001.

The IPA pathway analysis also demonstrated an activation of cell senescence pathway in the myofibroblasts of *Gli1^HHIPCKO^* (Fig. 2K). Consistent with the IPA results, scRNA-seq analysis showed an elevated expression of *Cdkn1a* (encoding p21), a senescent marker and mediator (*31*), in *Gli1^HHIPCKO^* myofibroblasts (Fig. 3A). The senescence-associated β-galactosidase activity analysis further confirmed the cell senescence in *Gli1^HHIPCKO^* lungs (Fig. 3B). Colocalization with SMA staining demonstrated cell senescence in the myofibroblasts of *Gli1^HHIPCKO^* lungs (fig. S5, A and B). Consistently, immunofluorescence staining for p21, along with SMA and CDH4, demonstrated cellular senescence in both ALMFs and DMFs of *Gli1^HHIPCKO^* lungs (Fig. 3, C to E). This was further confirmed by γ-histone H2AX (γ-H2AX) staining, another marker of cellular senescence (fig. S5, C to E). Notably, the stromal expression of *Cdkn2a* (encoding p16), another senescent marker, showed no significant difference (fig. S5F and tables S2 and S3), suggesting that p16 and p21 may independently mediate cellular senescence. Intriguingly, cellular senescence occurred not only in myofibroblasts but also in alveolar epithelial stem/progenitor cells (AT2s) (Fig. 3, F and G) and immune cells (fig. S5, G and H).

To further explore the effects of myofibroblast alteration on AT2s, we performed scRNA-seq analysis of AT2s of our *Gli1^HHIPCKO^* animals. Consistent with the p21 staining data, scRNA-seq analysis revealed a dramatic increase in the fraction of Cdkn1a^+^ AT2s in *Gli1^HHIPCKO^* mice (fig. S5, I and J). Furthermore, DEG analysis between AT2s of *Gli1^HHIPCKO^* versus the controls showed an increased expression of a set of procellular senescence and antiproliferation genes, including *Cdkn1a*, *Cdkn1b* (*32*), *B2m* (*33*), and *Ypel3* (*34*) (fig. S5K and table S4). Consistently, IPA pathway analysis of the up-regulated genes in the AT2s of *Gli1^HHIPCKO^* demonstrated a significantly activated cell senescence pathway (Fig. 3H). AT2s of *Gli1^HHIPCKO^* animals also demonstrated other senescent features, including an increase in the presence of γ-H2AX and senescence-associated β-galactosidase (fig. S5, L to O). Cell senescence is inversely related to proliferation. Consistently, the cell number of AT2s significantly decreased in our *Gli1^HHIPCKO^* animals (fig. S5, P and Q). Furthermore, colocalization with Ki67, a proliferating marker, showed that the percentage of proliferative AT2s in *Gli1^HHIPCKO^* animals was significantly lower than in the controls (fig. S5, P and R). These results suggest a non–cell-autonomous signaling from *Gli1^HHIPCKO^* stromal cells induces epithelial stem/progenitor cell senescence.

To explore upstream regulators driving AT2 senescence, we conducted IPA analysis of the AT2 DEGs (*Gli1^HHIPCKO^* versus control). IGF1 emerged as one of the top predicted upstream regulators in *Gli1^HHIPCKO^* AT2s (Fig. 3I), suggesting that stromal Hh activation affects AT2s via IGF1. To determine the effect of Hh activation on the alveolar niche in vitro, we used a mouse model (*R26R^SmoM2/+^*) in which Hh signaling can be activated in the isolated lung stromal cells by infecting with Cre-expressing adenoviruses. We then cocultured the AT2s of postnatal mice together with the Hh-activated stromal cells in our organoid system, resulting in a significant reduction of organoid growth (Fig. 3, J to L), and an increased expression of p21, compared with the control (Fig. 3, M and N). Blocking IGF1 signaling by adding a neutralizing anti-IGF1 antibody rescued organoid growth and suppressed p21 expression in AT2s (Fig. 3, J to N). These findings demonstrate that stromal Hh activation induced by *Hhip* deletion causes cellular senescence, both in stromal and epithelial stem/progenitor cells, in part via IGF1.

### Loss of HHIP induces BPD phenotype via stromal IGF1

**Fig. 4.**
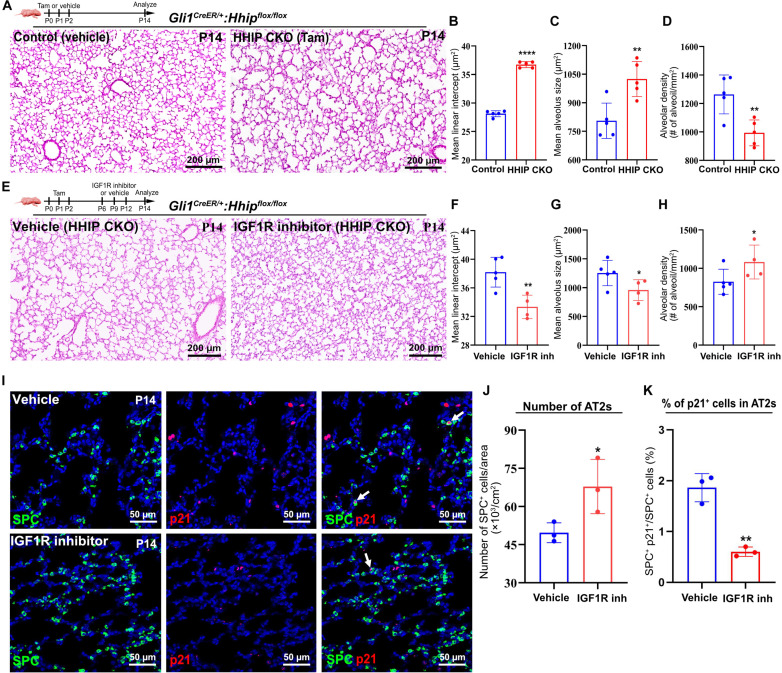
*Hhip* deletion causes BPD that can be attenuated by inhibiting IGF1 signaling. (**A**) H&E images of the lungs of *Hhip*-deleted and control mice. (**B** to **D**) Quantification of MLI, mean alveolus size, and alveolar density of *Hhip*-deleted and control lungs. (**E**) H&E images of the lungs of *Hhip*-deleted mice treated with vehicle or IGF1R inhibitor. (**F** to **H**) Quantification of MLI, mean alveolus size, and alveolar density of *Hhip*-deleted mice treated with vehicle or IGF1R inhibitor. (**I**) IF analysis of SPC and p21 in the lungs of *Hhip*-deleted mice treated with vehicle or IGF1R inhibitor. Arrow: p21^+^ SPC^+^ cells. (**J** and **K**) Quantification of the number of AT2s and percentage of p21^+^ AT2s in *Hhip*-deleted mice treated with vehicle or IGF1R inhibitor. Each data point represents one mouse [(B) to (D), (F) to (H), and (K)] of an individual experiment. Data are expressed as mean ± SD. *P < 0.05, **P < 0.005, and ****P < 0.0001.

Altered myofibroblasts and alveolar stem/progenitor cell senescence have been associated with BPD, and a recent genome-wide association study has implicated HHIP in human BPD susceptibility (*12*, *17*, *35*). To determine the effect of HHIP loss on alveolar morphogenesis, we used our *Gli1^HHIPCKO^* animals. Inducible deletion of *Hhip* caused a significant simplification of alveoli compared with controls at P14, as characterized by an increase in mean linear intercept (MLI) length and airspace along with reduced density of alveoli (Fig. 4, A to D), which are hallmarks of BPD.

HHIP regulates myofibroblast transition and alveolar niche via the Hh-IGF1 signaling axis (Figs. 2N and 3M). We then examined whether IGF1 signaling blockade can rescue the BPD phenotype in *Gli1^HHIPCKO^* mice. Histological assessment of alveolar morphometry revealed that IGF1R inhibitor–treated *Gli1^HHIPCKO^* animals had reduced MLI and airspace, along with increased alveolar density (Fig. 4, E to H). Furthermore, immunofluorescence analysis of IGF1R inhibitor–treated *Gli1^HHIPCKO^* lungs demonstrated an increased cell number in AT2s and a lower percentage of p21^+^ cells (Fig. 3, I to K). Together, these data suggest that excessive IGF1 secreted from myofibroblasts is both necessary and sufficient to drive BPD changes in our mouse model.

### Aberrant activation of Hh-IGF1 signaling in human BPD and the BPD animal model

**Fig. 5.**
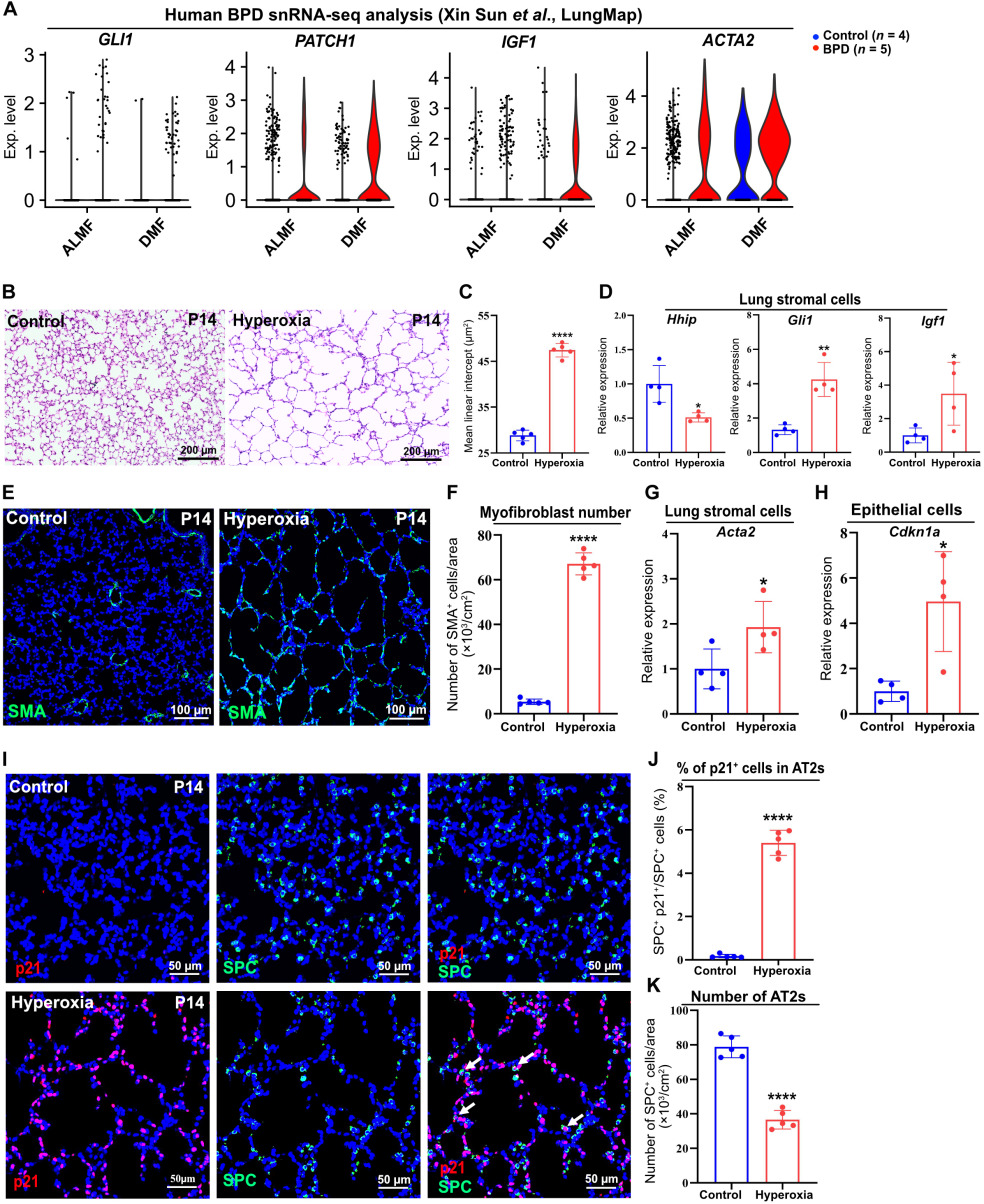
Overactivation of Hh-IGF1 signaling in BPD. (**A**) snRNA-seq analysis of *GLI1*, *PATCH1*, *IGF1*, and *ACTA2* expression in ALMFs and DMFs of human BPD. (**B**) H&E images of the lungs of hyperoxia-treated and control mice. (**C**) Quantification of MLI of hyperoxia-treated and control lungs. (**D**) qPCR analysis of *Hhip*, *Gli1*, and *Igf1* expression in the lung stromal cells isolated from hyperoxia-treated and control mice. (**E**) IF analysis of SMA in the lungs of hyperoxia-treated and control mice. (**F**) Histology quantification of the number of myofibroblasts of hyperoxia-treated and control mice. (**G**) qPCR analysis of *Acta2* expression in the lung stromal cells isolated from hyperoxia-treated and control mice. (**H**) qPCR analysis of *Cdkn1a* expression in the lung epithelial cells isolated from hyperoxia-treated and control mice. (**I**) IF analysis of p21 and SPC in the lungs of hyperoxia-treated and control mice. Arrow: p21^+^ SPC^+^ cells. (**J** and **K**) Quantification of the percentage of p21^+^ AT2s and number of AT2s in hyperoxia-treated and control mice. Each data point represents one mouse [(C), (D), (F) to (H), (J), and (K)] of an individual experiment. Data are expressed as mean ± SD. *P < 0.05, **P < 0.005, and ****P < 0.0001.

In the lung tissues of human BPD, cellular senescence has been consistently observed (*36*, *37*). To further explore potential alterations in Hh-IGF1 signaling within the lung stromal cells of human BPD, we reanalyzed publicly available single-nucleus RNA sequencing (snRNA-seq) data from LungMAP, originally generated by Sun’s group (*38*). Our analysis revealed overactivation of Hh signaling in human BPD, evidenced by increased expression of Hh signaling target genes *GLI1* and *PTCH1* in both ALMFs and DMFs, compared with controls (Fig. 5A). Furthermore, we observed up-regulation of *IGF1* and *ACTA2* expression in human BPD (Fig. 5A). These findings suggest that aberrant activation of Hh-IGF1 signaling may contribute to BPD pathogenesis in humans.

To mimic human BPD in preterm infants, we used a hyperoxia exposure neonatal mouse model (*39*). Histological analysis of lungs exposed to 80% O_2_ demonstrated a significant alveolar simplification compared with controls in normoxia (21% O_2_), as characterized by increased MLI and airspace (Fig. 5, B and C). qPCR analysis of the isolated lung stromal cells of hyperoxia-exposed mice showed a significant decrease in *Hhip* expression (Fig. 5D). Consistently, we observed aberrant Hh activation in hyperoxia-exposed lungs, indicated by up-regulation of *Gli1* expression (Fig. 5D). Of note, *Igf1* expression significantly increased in hyperoxia-exposed mice (Fig. 5D). In line with previous studies (*5*), we observed an increased presence of SMA^+^ myofibroblasts in the alveoli of hyperoxia-exposed mice at P14 (Fig. 5, E and F), which was further confirmed by qPCR analysis showing elevated *Acta2* expression in the lung stromal cells of these mice compared with controls (Fig. 5G). qPCR analysis of the epithelial cells from hyperoxia-exposed mice showed up-regulated *Cdkn1a* expression (Fig. 5H). Immunofluorescence staining for p21 revealed an increase in senescent cells in the lungs of hyperoxia-treated mice (Fig. 5 I). Colocalization with SPC indicated that the proportion of senescent cells among AT2s increased as well, while the number of AT2s decreased (Fig. 5, I to K). Thus, these data suggest that hyperoxia-exposed mice serve as a robust model for human BPD, with Hh-IGF1 signaling overactivation playing a critical role in BPD pathogenesis.

### Blockade of Hh-IGF1 signaling attenuates BPD

**Fig. 6.**
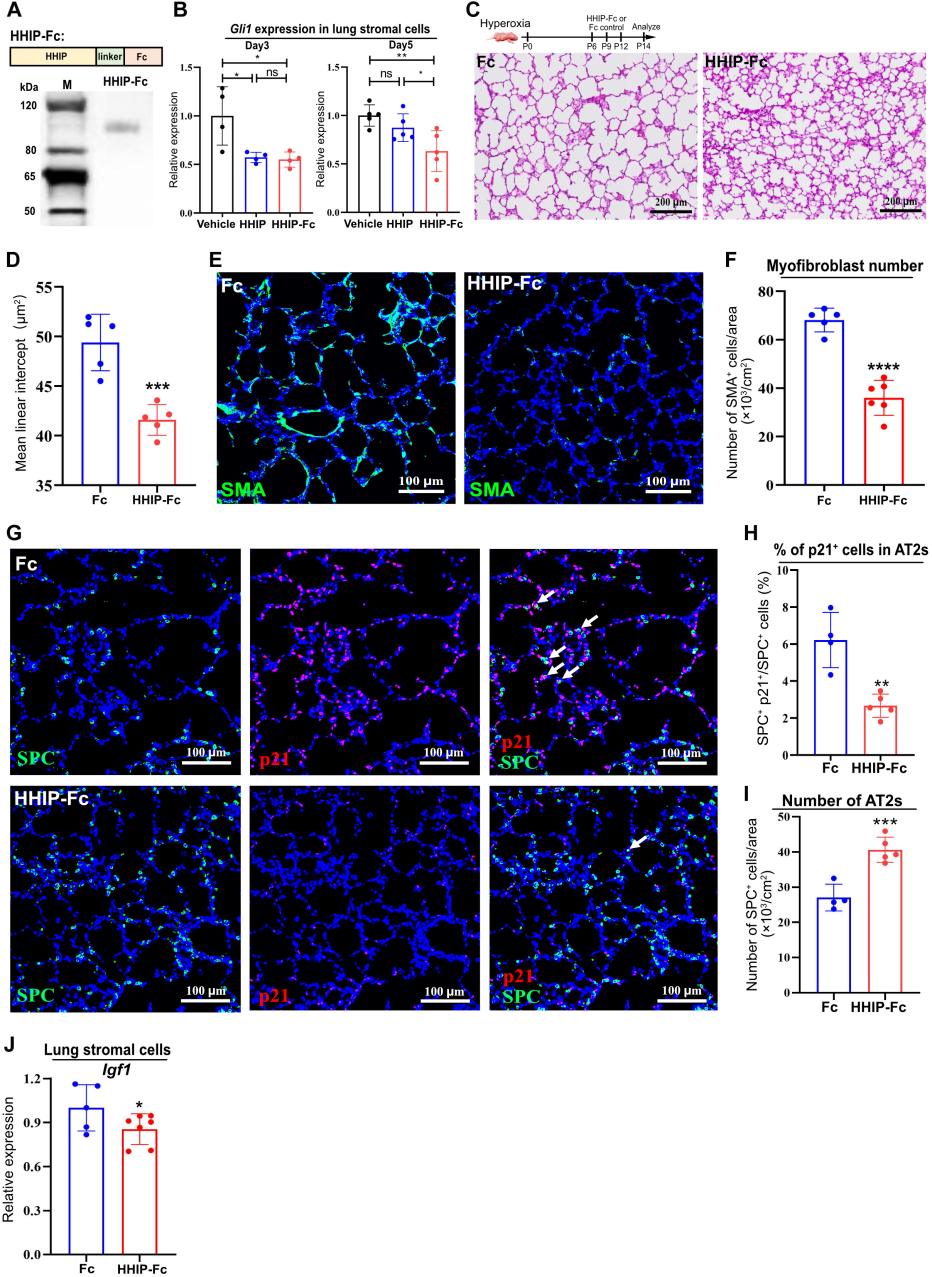
HHIP-Fc protein alleviates hyperoxia-induced BPD phenotypes. (**A**) Design strategy for HHIP-Fc recombinant protein and its analysis by Western blotting. (**B**) qPCR analysis of *Gli1* expression in the lung stromal cells isolated from neonatal mice 3 and 5 days after one dose of HHIP or HHIP-Fc treatment. (**C**) H&E images of hyperoxia-exposed lungs, treated with HHIP-Fc or Fc fragment control. (**D**) MLI quantification of hyperoxia-exposed lungs, treated with HHIP-Fc or Fc. (**E** and **F**) IF analysis and quantification of myofibroblasts in hyperoxia-exposed lungs, treated with HHIP-Fc or Fc. (**G** to **I**) IF analysis and quantification of AT2 number and p21^+^ AT2 percentage in hyperoxia-exposed lungs, treated with HHIP-Fc or Fc. Arrow: p21^+^ SPC^+^ cells. (**J**) qPCR analysis of *Igf1* expression in the lung stromal cells isolated from hyperoxia-exposed mice, treated with HHIP-Fc or Fc. Each data point represents one mouse [(B), (D), (F), and (H) to (J)] of an individual experiment. Data are expressed as mean ± SD. *P < 0.05, **P < 0.005, ***P < 0.0005, and ****P < 0.0001.

Currently, there is still no specific and effective cure for BPD. Here, we sought to examine whether administration of HHIP protein can attenuate hyperoxia-induced BPD. Therapeutic Fc-fusion proteins, linking the Fc region of immunoglobulin G (IgG) antibody to a desired protein, are successful biopharmaceuticals due to their long-lasting effects (*40*). To determine the therapeutic effect of Fc-fused HHIP protein on BPD, we generated a recombinant HHIP-Fc protein with truncated HHIP fused with Fc fragment (Fig. 6A). We first tested the efficacy of HHIP-Fc protein in vivo. qPCR analysis of the lung stromal cells from neonatal mice treated with a single dose of vehicle, HHIP, and HHIP-Fc revealed that both HHIP and HHIP-Fc significantly inhibited the expression of *Gli1* 3 days postadministration. On the fifth day, HHIP-Fc continued to effectively suppress *Gli1* expression, while HHIP no longer had a significant effect, indicating that HHIP-Fc had a more sustained inhibitory impact on Hh signaling in vivo (Fig. 6B). We then administered HHIP-Fc to postnatal mice exposed to hyperoxia. Histologically, hyperoxia-exposed animals treated with HHIP-Fc demonstrated a significant improvement in alveolar structure formation, with decreased airspace and MLI, compared with Fc controls (Fig. 6, C and D). To assess the therapeutic impact of HHIP-Fc more thoroughly, we conducted analyses on myofibroblasts and AT2s. Immunofluorescence staining for SMA revealed that HHIP-Fc administration led to a marked reduction in the SMA^+^ myofibroblasts within the lungs of mice subjected to hyperoxia (Fig. 6, E and F). Moreover, treatment with HHIP-Fc significantly diminished the population of senescent cells in the mice, a reduction also observed within the AT2s, alongside a concomitant rise in the number of AT2s (Fig. 6, G to I). qPCR analysis of the lung stromal cells of HHIP-Fc–treated mice showed that HHIP-Fc effectively suppressed the expression of *Igf1* (Fig. 6J). These data indicate that BPD is largely attributed to Hh activation, and supplementing HHIP-Fc protein can alleviate the disease severity, highlighting the promising clinical potential of HHIP-Fc for treating BPD.

### Adult mice with *Hhip* haploinsufficiency develop emphysema, ameliorated by HHIP-Fc administration

**Fig. 7.**
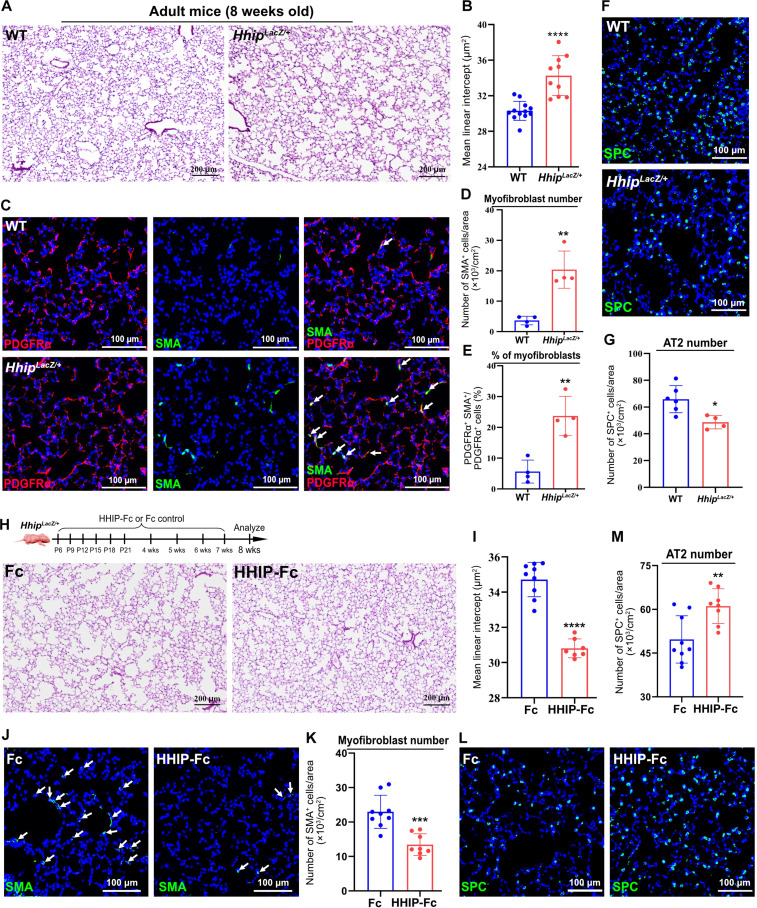
HHIP-Fc administration alleviates emphysema in adult mice with *Hhip* haploinsufficiency. (**A** and **B**) H&E images and MLI quantification of the lungs from adult *Hhip^LacZ/+^* and WT mice. (**C**) IF analysis of PDGFRα and SMA in the lungs of adult *Hhip^LacZ/+^* and WT mice. Arrow: SMA^+^ PDGFRα^+^ cells. (**D** and **E**) Quantification of the number and percentage of myofibroblasts in adult *Hhip^LacZ/+^* and WT mice. (**F** and **G**) IF analysis of SPC and quantification of AT2s in the lungs of adult *Hhip^LacZ/+^* and WT mice. (**H** and **I**) H&E images and MLI quantification of the lungs from adult *Hhip^LacZ/+^* and WT mice treated with HHIP-Fc or Fc. (**J** and **K**) IF analysis and quantification of myofibroblasts in the lungs of adult *Hhip^LacZ/+^* and WT mice treated with HHIP-Fc or Fc. Arrow: SMA^+^ cells. (**L** and **M**) IF analysis and quantification of AT2s in the lungs of adult *Hhip^LacZ/+^* and WT mice treated with HHIP-Fc or Fc. Each data point represents one mouse [(B), (D), (E), (G), (I), (K), and (M)] of an individual experiment. Data are expressed as mean ± SD. *P < 0.05, **P < 0.005, ***P < 0.0005, and ****P < 0.0001.

BPD is a major risk factor for COPD/emphysema, with *HHIP* implicated in both conditions (*15*–*17*, *20*). Therefore, we hypothesized that HHIP deficiency in neonates leads to alveolar malformation, which subsequently causes emphysema in adults. Histological analysis of adult *Gli1^HHIPCKO^* mice, in which *Hhip* was deleted at the neonatal stage, demonstrated increased airspace and MLI, indicative of an emphysema phenotype (fig. S6, A and B). To model the insufficient *HHIP* expression observed in human patients with COPD (*41*), we further used *Hhip* heterozygous (*Hhip^LacZ/+^*) mice. Consistently, histological analysis of adult *Hhip^LacZ/+^* lungs confirmed an emphysema phenotype, marked by increased airspace and MLI, compared with the wild-type (WT) controls (Fig. 7, A and B). Furthermore, immunofluorescence staining of PDGFRα and SMA revealed a significant increase in myofibroblasts within the alveoli of *Hhip^LacZ/+^* mice (Fig. 7, C to E). In addition, the cell number of AT2s was significantly reduced in *Hhip^LacZ/+^* lungs, compared with WT controls (Fig. 7, F and G). To determine whether BPD-associated emphysema can be prevented by correcting neonatal alveolar development, we administered HHIP-Fc protein to *Hhip^LacZ/+^* mice at the neonatal stage and analyzed lung morphology at 8 weeks of age. Histologic assessment of alveolar morphometry revealed that HHIP-Fc–treated mice had reduced alveolar space and MLI (Fig. 7, H and I). In addition, HHIP-Fc treatment significantly reduced the number of myofibroblasts in the alveoli (Fig. 7, J and K) and increased the number of AT2s (Fig. 7, L and M). Together, these data suggest that *Hhip* deficiency is sufficient to drive emphysematous changes, and HHIP-Fc can attenuate BPD-associated emphysema.

## DISCUSSION

Precise regulation of the timing window period for SMA^+^ myofibroblast formation and disappearance is essential for alveologenesis. Our study demonstrates how HHIP, through restricting Hh activation, can modulate the dynamic changes of myofibroblasts and protect alveologenesis, providing a theoretical rationale for the clinical trial of HHIP-Fc recombinant protein for BPD and BPD-associated emphysema. Furthermore, we find that IGF1, a secreted niche factor, is regulated by HHIP during alveologenesis. Excessive IGF1 alters the myofibroblast transition and induces epithelial stem/progenitor senescence, which can be rescued by administering HHIP-Fc protein.

Hh signaling is a key developmental pathway that coordinates the cross-talk among a group of cells and its tight regulation is required for organ morphogenesis (*42*–*44*). In alveologenesis, it has been reported that Hh-responsive (Gli1^+^) myofibroblasts were present in all alveolar walls during the first week of postnatal life, after which the cell number of Gli1^+^ cells dramatically decreased (*27*). Lineage-tracing analysis showed that those GLI1-lineage cells labeled at P5 and P6 were still widely present in the alveoli of P11 lungs (*45*). The findings indicate that approximately 1 week postnatally, Hh signaling pathway undergoes a progressive decline, a process that appears to be crucial for the transition of myofibroblasts and for the maturation of alveoli. However, the questions remain as to how the dynamic Hh signaling is regulated in vivo and by which mechanism Hh signaling controls myofibroblast transition. Our data show an increased expression of stroma-derived HHIP, the antagonist of Hh, that restricts Hh activation and drives myofibroblast transition during alveologenesis. We have previously shown that HHIP suppresses tissue inflammation and maintains adult lung homeostasis (*24*). Our current data further demonstrate that HHIP also protects alveologenesis and promotes myofibroblast transition by restricting the Hh-IGF1 signaling axis. These findings indicate that HHIP is essential for both lung development and its ongoing maintenance.

Senescence is often considered as an age-associated pathological state in which senescent cells undergo cell cycle arrest, and they accumulate overtime with aging (*31*). Numerous studies have shown that IGF1 is associated with cellular senescence in aging, and life extension has been achieved in animal models by suppressing IGF1 signaling (*46*–*49*). Our study demonstrates that excessive stromal IGF1 in the *Hhip*-deleted and hyperoxia-exposed mice induces cell senescence in different cell types, including myofibroblasts and AT2s, in postnatal mice. Consistent with our findings, previous studies have shown that deficiency in IGF1R confers protection against hyperoxia-induced lung injury and radiation-induced AT2 senescence in mice (*50*, *51*). In addition, IGF1 has been demonstrated to induce AT2 senescence (*52*). Using both immunofluorescence staining and RNA sequencing, we observed up-regulated expression of p21/*Cdkn1a* in AT2s of *Hhip*-deleted mice. However, the magnitude of this increase differed substantially between the two methods, likely attributed to the lower sensitivity of immunofluorescence staining. Furthermore, we detected a reduced proportion of Ki67^+^ AT2s in *Hhip*-deleted mice, although baseline levels in WT mice were already minimal. We speculate that Ki67 staining captures only cells actively proliferating at the time of tissue fixation, providing a transient snapshot of proliferation. To better track proliferative AT2 cells during alveologenesis, administering Edu or BrdU (which labels cells that have undergone DNA synthesis) might reveal a higher proportion of proliferating cells. Notably, a prior study demonstrated that while Ki67^+^ T cells remain relatively constant across time points, BrdU labeling reveals a significant temporal increase in proliferative T cells (*53*).

Both p21 and p16 are widely recognized as markers of senescent cells and are known to mediate key functions in these cells. However, there is limited understanding of whether p21 and p16 are differentially expressed across distinct senescent cell populations and whether their functions diverge. A recent scRNA-seq study demonstrated that p21 and p16 expression exhibits significant heterogeneity across various cell types, each with distinct secretomes, often with minimal coexpression (*54*). In addition, targeted clearance of p21^+^, but not p16^+^, senescent cells effectively prevented radiation-induced osteoporosis and reduced marrow adiposity (*55*). In our current work, deletion of *Hhip* specifically up-regulated p21 expression without altering p16 levels in stromal cells. Intriguingly, while our prior work established that p16^+^ senescent stromal cells promoted airway epithelial proliferation during injury repair (*56*), we now demonstrated that p21^+^ senescent stromal cells induced by *Hhip* deletion suppressed AT2 proliferation. These findings underscore a functional dichotomy between p21 and p16 in senescent cell biology, suggesting that their roles may be context and cell type dependent.

Without targeted preventive therapies, BPD remains the most common complication in preterm infants and is increasing in prevalence (*57*, *58*). Having advanced supportive medical care, most infants with BPD can survive, but they will face an increased risk of COPD/emphysema in adulthood, exhibiting similar phenotype to BPD (*16*). Notably, HHIP has been implicated in both BPD and COPD/emphysema (*17*, *20*). Our previous study demonstrated a protective role of HHIP against COPD/emphysema acute exacerbation. Here, we demonstrate that HHIP protects alveologenesis and mitigates BPD by constraining the overactivation of Hh-IGF1 signaling. We acknowledge the presence of inconsistent findings regarding IGF1 and IGF1R expression levels in the context of BPD. Low serum IGF1 levels have been observed in BPD infants (*59*, *60*), suggesting a systemic IGF1 deficiency that may increase susceptibility to the disease. In contrast, elevated IGF1 and IGF1R levels have been consistently reported in lung tissues of BPD infants (*61*–*63*). Our analysis of snRNA-seq data from the LungMAP database further confirmed the up-regulation of IGF1 in lung tissues of BPD infants. In hyperoxia-induced BPD animal models, elevated IGF1 and IGF1R expression levels in lung tissues have also been reported by multiple independent studies, consistent with our findings (*64*–*66*). This disparity between systemic and local IGF1 expression likely reflects the distinct roles of IGF1 in systemic circulation versus pulmonary tissue during BPD progression. Of note, our findings demonstrate that HHIP-Fc protein alleviates hyperoxia-induced BPD, with consistent reductions in MLI. While *Igf1* was significantly down-regulated after HHIP-Fc treatment, its variability was higher than MLI. This may reflect differing temporal dynamics, as MLI indicates long-term effects, whereas *Igf1* qPCR captures a snapshot of the response at a single time point. In addition, while *Igf1* is a key HHIP-regulated effector, it may not be the sole mediator. In *Hhip* knockout neonates, other growth factors such as *Hgf* and *Wnt2* showed downward trends, although these changes were not statistically significant. Nevertheless, both factors are critical for alveologenesis (*67*, *68*). In addition, our data show that administering HHIP-Fc protein during the alveolar development stage significantly alleviates BPD-associated emphysema. Our study highlights that antagonizing the Hh-IGF1 signaling pathway is a potential targeted therapeutic strategy for BPD and BPD-associated COPD/emphysema.

One limitation of our study is that we have not addressed the role of myofibroblasts that become SMA-negative and persist in the alveoli. Future studies are required to understand the function of SMA^+^ myofibroblast–derived cells in regulating the continued alveologenesis. It is also crucial to investigate whether these cells persist in the adult lung and, if so, to determine their involvement in maintaining alveolar homeostasis or contributing to chronic lung diseases. We recognize that, in addition to HHIP expression levels, the transmission and activation of Hh signaling are regulated by multiple factors. These include other negative regulators like suppressor of fused, ligand, and receptor expression levels, as well as the presence of primary cilia on receiving cells (*69*, *70*). A more comprehensive understanding of the regulatory network controlling Hh signaling in the lung will require further investigation into these factors. While we acknowledge that the pathogenesis of human BPD and emphysema is likely multifactorial, our study proposes a model in which HHIP deficiency disrupts stromal function, thereby impairing alveologenesis and contributing to the phenotypes of BPD and BPD-associated emphysema.

## MATERIALS AND METHODS

### Animal studies

*Hhip^flox/flox^* line was generated as we previously described (*24*). Generation and genotyping of the *Gli1^CreER^*, *Gli1^LacZ^*, *Hhip^LacZ^*, *R26R ^SmoM2^*, and *R26R ^tdTomato^* lines were performed as previously described by The Jackson Laboratory. *Sftpc^CreER^* line was generously provided by Chapman *et al.* (*71*). *Acta2^DreER^* and *R26R^RSR-tdTomato^* mice were generously provided by B. Zhou (*72*). All experiments were performed according to the institutional ethical guidelines on animal care and approved by the Institute Animal Care and Use Committees at Shanghai Institute of Materia Medica and Zhongshan Institute for Drug Discovery (no. 2022-08-WCQ-01).

### Animal treatment

For activating CreER and DreER, tamoxifen (Tam; catalog no. T006000; TRC) was dissolved in corn oil and administered intraperitoneally at 100 mg/kg body weight per day for 3 days. Newborn mouse pups from dams that delivered on the same day were randomized at day of birth (postnatal day 0) and divided to equal-sized litters. Following randomization, mouse cages were either maintained in room air (normoxia, 21% O_2_) or in hyperoxia (80% O_2_) from P0 until the day of harvest. In addition to the continuous hyperoxia treatment, mice were received intraperitoneal injections of 1 mg/kg body weight Fc or HHIP-Fc on P6, P9, and P12. *Hhip^LacZ/+^* mice were received intraperitoneal injections of 1 mg/kg body weight Fc or HHIP-Fc on days P6, P9, P12, P15, P18, and P21, as well as at 4, 5, 6, and 7 weeks of age. HHIP-Fc protein was designed by C.W. and made by DetaiBio. For IGF1R inhibitor treatment, *Gli1^CreER/+^:Hhip^flox/flox^* mice were administered NVP-ADW742 (catalog no. SML1921; Sigma-Aldrich) at a dosage of 5 mg/kg body weight on days P6, P9, and P12.

### Histology and immunofluorescence

The right ventricle of the mice was perfused with 1 to 3 ml of phosphate-buffered saline (PBS) and the lungs were inflated with 4% paraformaldehyde (PFA) in PBS and then fixed in 4% PFA overnight at 4°C. After fixation, the lungs were washed with cold PBS four times for 30 min each at 4°C and then dehydrated in a series of increasing ethanol concentration washes (30, 50, 70, 95, and 100%) for a minimum of 2 hours per wash. The dehydrated lungs were incubated with xylene for 1 hour at room temperature (RT) and then with paraffin at 65°C for 90 min twice, and then embedded in paraffin. The lungs were sectioned at 8 μm on a microtome. For frozen optimal cutting temperature (OCT) embedding, lungs were inflated and fixed with 4% PFA for 2 hours at 4°C, washed with PBS four times for 30 min each at 4°C, and embedded in OCT after 30% sucrose incubation. For histologic analysis of organoids, transwells were fixed in 4% PFA overnight at 4°C, then washed in PBS overnight at least three times. Transwells were cut and embedded in OCT after 30% sucrose incubation, then 8-μm sections were cut on a cryostat.

For immunofluorescent staining, paraffin sections were incubated in xylene for 10 min twice, then rehydrated in ethanol washes (100% twice, 95, 70, and 50% ethanol) for 5 min each. OCT embedded slides were fixed in 4% PFA at RT for 10 min, then washed three times with PBS. For both paraffin and OCT embedded slides, antigen retrieval (catalog no. BRR2004CLX; Biocare Medical) was performed for 30 min at 95°C. Slides were washed with 0.1% Tween 20 in PBS (PBST) three times for 5 min. Slides were then incubated in blocking buffer (3% donkey serum in PBST) for at least 1 hour, then incubated overnight in primary antibodies in 1% donkey serum in PBST at 4°C. The following primary antibodies were used for mouse tissue: goat anti-HHIP (catalog no. AF1568; R&D Systems; used 1: 250), rat anti-CDH4 (catalog no. MRCD5; DSHB; used 1: 20), rabbit anti-CDH4 (catalog no. BS40099; Bioworld; used 1: 100), rabbit anti-SMA (catalog no. ab5694; Abcam; used 1:400), mouse anti-SMA (catalog no. AB7817; Abcam; used 1:500), goat anti-tdTomato (catalog no. AB8181-200; OriGene; used 1:500), rabbit anti-RFP (catalog no. 600-401-379; Rockland; used 1:500), rabbit anti-SPC (catalog no. AB3786; Millipore Sigma; used 1:2000), rat anti-Ki67 (catalog no. 14-5698-82; Thermo Fisher Scientific; used 1:100), rabbit anti-γ-H2A (catalog no. 9718T; Cell Signaling Technology; used at 1:250), rat anti-p21 (catalog no. ab107099; Abcam; used 1:100), rat anti–DC-LAMP (catalog no. DDX0191P-100; Novus; used 1:250), goat anti-PDGFRα (catalog no. AF1062; R&D Systems; used 1:200), and rabbit anti-PDGFRα (catalog no. 3174; Cell Signaling Technology; used 1:200). Slides were washed with PBST and then incubated for 1 hour at RT in secondary antibodies diluted in PBST. The following secondary antibodies were used at 1:500: donkey anti-rabbit IgG Alexa Flour 555 (catalog no. A-31572; Thermo Fisher Scientific), donkey anti-rabbit IgG Alexa Flour 647 (catalog no. A-31573; Thermo Fisher Scientific), donkey anti-goat Alexa Flour 555 (catalog no. A-21432; Thermo Fisher Scientific), donkey anti-goat Alexa Flour 647 (catalog no. A-21447; Thermo Fisher Scientific), donkey anti-mouse Alexa Flour 488 (catalog no. A-21202; Thermo Fisher Scientific), and donkey anti-rat Alexa Flour 647 (catalog no. 712-605-153; Jackson ImmunoResearch). DAPI (4′,6-diamidino-2-phenylindole; 0.2 μg/ml) (catalog no. XW287189031; Sinopharm) was added for 5 min, and slides were then washed with PBS three times for 5 min and Fluorsave to maintain fluorescence. For X-gal staining, frozen sections were allowed to dry at RT up to 1 hour and rinsed with PBS for 5 min three times, then stained with X-gal staining solution [consisting of X-gal (catalog no. V900468; Sigma-Aldrich), potassium ferricyanide, potassium ferrocyanide, MgCl_2_, NP40, dimethylformamide, and PBS] at 37°C for 2 hours. After staining, sections were washed with PBS three times for 5 min. The senescence β-galactosidase staining was performed according to the manufacturer’s instructions (catalog no. 9860S; Cell Signaling Technology).

### RNA in situ hybridization

OCT-embedded lung sections were used for RNA in situ hybridization with the RNAscope Multiplex Fluorescent Reagent Kit version 2 (catalog no. 323100; Advanced Cell Diagnostics) according to the manufacturer’s instructions. RNA probes for *Gli1* (catalog no. 311001; Advanced Cell Diagnostics), *Gli2* (catalog no. 405771; Advanced Cell Diagnostics), and *Igf1* (catalog no. 443901; Advanced Cell Diagnostics) were used. Following the completion of RNA in situ hybridization, immunostaining was performed after blocking, as described above.

### Lung digestion and flow cytometry

The whole mouse lung was dissected and tracheally perfused with a digestion cocktail of dispase II (catalog no. 17105041; Thermo Fisher Scientific; used 15 U/ml), collagenase type I (catalog no. 17100017; Thermo Fisher Scientific; used 500 U/ml), and deoxyribonuclease I (catalog no. DN25; Sigma-Aldrich; used 50 U/ml), and removed from the chest. The lung was further diced with a razor blade and incubated in digestion cocktail for 20 min at 37°C with continuous shaking. The mixture was then washed with sorting buffer [2% fetal bovine serum (FBS; catalog no. 10091148; Thermo Fisher Scientific) and 1% antibiotic-antimycotic (catalog no. 15240062; Thermo Fisher Scientific) in Dulbecco’s minimum essential medium (DMEM; catalog no. 31053028; Thermo Fisher Scientific)]. The mixture was passed through a 70-μm cell strainer and resuspended in red blood cell lysis buffer (catalog no. R7757; Sigma-Aldrich), then passed through a 40-μm cell strainer.

For fluorescence-activated cell sorting (FACS), cell suspensions were incubated with the appropriate conjugated antibodies in sorting buffer for 30 min at 4°C and washed with sorting buffer. FACS was performed on a BD FACSAria II using FACSDiva software. Doublets and dead cells were excluded on the basis of forward and side scatter and DAPI, respectively. Immune and endothelial cells were excluded using CD45 (BV421; catalog no. 563890; BD; used 1:200) and CD31 (Alexa Fluor 488; catalog no. 102414; BioLegend; used 1:200), respectively. Epithelial cells were gated base on CD326 (APC; catalog no. 17-5791-82; Invitrogen; used 1:200). Analysis was performed using FlowJo version 10 software. In addition, lung stromal cells were also enriched using the EasySep Mouse Streptavidin RapidSpheres Isolation Kit (catalog no. 19860; Stem Cell Technologies) and immune, endothelial, and epithelial cells were excluded using CD45 (catalog no. 103104; BioLegend; used 2 μg/ml), CD31 (catalog no. 102404; BioLegend; used 2 μg/ml), and CD326 (catalog no. 118204; BioLegend; used 2 μg/ml). Cells were sorted into FACS buffer.

For FACS analysis of SMA expression, cells were staining for CD45 (BV421; catalog no. 563890; BD; used 1:200), CD31 (BV421; catalog no. 404-0311-82; Invitrogen; used 1:200), CD326 (BV786; catalog no. 740958; BD; used 1:200), and PDGFRα (APC; catalog no. 17-1401-81; Invitrogen; used 1:200). After surface marker staining, the cells were fixed and permeabilized using the Foxp3 Staining Buffer Set (catalog no. 00-5523-00; eBioscience), followed by SMA staining (AF488; catalog no. 53-9760-80; Invitrogen; used 1:200). Zombie NIR (catalog no. 423105; BioLegend) was used to exclude dead cells.

### Single-cell capture and sequencing

For scRNA-seq, all live lung stromal cells (CD45^−^/CD31^−^/CD326^−^) and epithelial cells (CD45^−^/CD31^−^/CD326^+^) were sorted from the *Gli1^creER/+^*: *Hhip^flox/flox^* mouse (mutant), which was administered daily Tam injections for 3 days starting at birth and analyzed at P14. The control mouse (*Gli1^creER/+^*: *Hhip^flox/flox^*) received daily oil injections for 3 days starting at birth. Lung stromal cells were isolated on the basis of forward and side scatter, DAPI, CD31, CD45, and CD326 exclusion. Epithelial cells were isolated on the basis of DAPI, CD31 and CD45 exclusion, and CD326-positive selection. To minimize sequencing costs, stromal cells and epithelial cells from the same mouse were combined in a 3:1 ratio of stromal cells to epithelial cells before library preparation and sequencing. The sorted cells were then loaded onto a single lane per sample into the Chromium Controller to produce gel bead-in emulsions (GEMs). GEMs underwent reverse transcription for RNA barcoding and cDNA amplification, with the library prepped using the Chromium Next GEM Single Cell 5’ Kit version 2. Each sample was sequenced in one lane of the Illumina NovaSeg 6000. A total of 12,350 cells were captured from the mutant sample, with 21,437 genes detected. The mean reads per cell was 35,450, the median unique molecular identifier (UMI) counts per cell was 3720, and the median number of genes per cell was 1699. In the control sample, 10,999 cells were captured, with 21,254 genes detected. The mean reads per cell was 32,846, the median UMI counts per cell was 3531, and the median number of genes per cell was 1564.

### Cell culture

Stromal cells were isolated from lungs dissected from P12 or adult mice, which were digested as described above, and subsequently cultured on cell culture plates in DMEM/F12 (catalog no. 11330057; Thermo Fisher Scientific) with 10% FBS and 1% antibiotic-antimycotic. Media was refreshed every other day and primary lung stromal cells were maintained for no more than five passages. For SHH stimulation, the confluent fibroblasts were cultured in 0.5% FBS with 1% antibiotic-antimycotic, and recombinant SHH (catalog no. 464-SH-025/CF; R&D) was added at 50 ng/ml with or without recombinant HHIP (2.5 mg/ml; catalog no. 1568-HP-050/CF; R&D) or with or without NVP-ADW742 (0.5 μg/ml). For IGF-1 treatment, the confluent fibroblasts were cultured in 0.5% FBS with 1% antibiotic-antimycotic, and recombinant murine IGF1 (catalog no. 250-19; PeproTech) was added at 50 ng/ml. Then, RNA was extracted from the cells after 3 days. The *R26R ^SmoM2/+^* mouse stromal cells were treated with Adv-empty or Adv-Cre for 72 hours. When used for organoid assays, Adv treatment occurred for 72 hours immediately before coculture with AT2s.

### Organoid assay

For the organoid assay, tdT+ AT2s were sorted from Tam-injected *Sftpc^creER/+^*: *R26R^tdTomato^* P7 mice. AT2s and lung stromal cells were cocultured (10,000 AT2s: 30,000 stromal cells per well) in a modified mouse tracheal epithelial cell (MTEC) medium diluted 1:1 in growth factor-reduced Matrigel (catalog no. 354230; Corning). The modified MTEC culture medium (catalog no. C-21170; PromoCell) is composed of small airway basal media with selected components from small airway epithelial cell (SAGM) bullet kit including insulin, transferrin, bovine pituitary extract, retinoic acid, and human epidermal growth factor. Ten percent FBS and 1% antibiotic-antimycotic were also added to the medium. Cell suspension-Matrigel mixture was placed in a transwell and incubated in growth medium with 10 μM ROCK inhibitor (catalog no. 72252; Stem Cell Technologies) in a 24-well plate for 48 hours, after which the medium was replenished every other day (lacking ROCK inhibitor). Each experimental condition was performed in triplicates. Where applicable, anti-IGF1 (catalog no. P157G; PeproTech; used at 1 μg/ml) or goat IgG (catalog no. 500-G00; PeproTech; used at 1 μg/ml) were added to the medium after 72 hours and replenished in every medium change. Organoids were assayed after 14 days.

### Quantitative PCR

Total RNA was obtained from cultured or freshly sorted cells using the FastPure Cell/Tissue Total RNA Isolation Kit version 2 (catalog no. RC112; Vazyme), following the manufacturer’s protocol. cDNA was synthesized from total RNA using the HiScript III RT SuperMix (catalog no. R323, catalog no. R323; Vazyme). qPCR was performed using the SYBR Green system (catalog no. Q711; Vazyme). Primers are listed in table S5. Relative gene expression levels after qPCR were defined using the ∆∆Ct method and normalizing to glyceraldehyde-3-phosphate dehydrogenase.

### Immunofluorescence image quantification

Sections were imaged for quantification on the Olympus FV3000 or Zeiss LSM 980 Confocal Microscope. At least three samples per genotype/condition were used, and at least five randomly selected sections were chosen for each sample. Cell counts for stained cells were performed in Fiji using the “Cell Counter” plug-in. For quantification by area, the image was first converted to 8 bit and the “Measure” function was used. For all analyses, performer was blinded to the specimen genotype/condition during data collection and analysis. Results were averaged between each specimen and SDs were calculated per genotype/condition.

### MLI, airspace size, and alveolar density analysis

For alveolar morphometric analysis, lungs were processed according to the above protocol for paraffin embedded samples, with the exception of inflation with 4% PFA at a constant pressure of 25-cm H_2_O. The paraffin-embedded lung sections were stained by hematoxylin and eosin (H&E) for analyzing alveolar morphology metric. At least five randomly selected sections from each genotype were selected for analysis. The MLI was calculated as the linear sum of the lengths of all lines randomly drawn across the images, divided by the number of intersections between alveolar walls. A minimum of 1000 intercepts from 50 lines drawn across the lung in a randomized fashion were obtained for each lung, and the analysis was carried out on Fiji with the “Cell Counter” plug-in. The airspace size was measured with Fiji using the analyze particles tool. Images were first converted to 8 bit and inverted, and the “analyze particle” function was used with a set minimum of 50 μm^2^ and maximum of infinity to identify and quantify alveoli in the image. The average airspace size for each lung was quantified by dividing the total airspace by the number of alveoli. At least 3500 alveoli were measured for each lung. The alveolar density was the reciprocal of the airspace size. The airspace of airway and pulmonary vessels was excluded.

### Single-cell RNA-seq analysis

For the animal data, FASTQ files were run through CellRanger version 6.1.0 software with default settings for demultiplexing, aligning reads with STAR software to mm10, and counting UMIs. Seurat package version 3 in RStudio was used for downstream analysis (*73*). Low-quality cells were filtered (expressing fewer than 200 genes, >10% mitochondrial reads and >6000 unique gene counts). Principal components analysis was performed on log-normalized and scaled data using 2000 variable genes. The top 30 principal component analyses were used for clustering and visualized using the UMAP algorithm in the Seurat package. The lists of DEGs were identified with a Model-based Analysis of Single-cell Transcriptomics test. Pathway and upstream regulator analyses of gene lists containing significantly DEGs were done with IPA (Qiagen). The representation factor was calculated to represent the number of overlapping genes divided by the number of expected overlapping genes drawn from two independent groups, as calculated on nemates.org with a base value of 30,000 genes in the mouse genome. A representation factor of >1 indicates more overlapping genes than expected of two independent groups. GO enrichment analysis was performed using the PANTHER overrepresentation test and entering the top 100 signature genes in ALMFs and DMFs. The expression levels of *Igf1* and *Igf1r* across various cell types were analyzed using the Gene Expression Omnibus data: GSE180822 (*25*). For the human BPD snRNA-seq analysis, we reanalyzed age-matched (infant) publicly available data from LungMAP, originally generated by Sun *et al.* (*38*).

### Statistical analysis

All statistical analyses were performed in GraphPad Prism 8.0.2 using unpaired two-tailed Student’s *t* tests to determine the *P* value. Specific *P* values were labeled in the plots or figure legends, where significant values were *P* < 0.05. All data in graphs were presented as mean ± SD. No data were excluded from the analyses presented in this study. Three or more replicates were used for every study and specific n values were listed in the figure legends.
