# Supplementary material for: Hedgehog-interacting protein orchestrates alveologenesis and protects against bronchopulmonary dysplasia and emphysema
Source: Sci Adv. 2025 May 7;11(19):eadu2958. doi: 10.1126/sciadv.adu2958 (PMC12057671; doi:10.1126/sciadv.adu2958)
Supplement: Supplementary file 1 — Figs. S1 to S6 Legends for tables S1 to S5 [file sciadv.adu2958_sm.pdf]

Science Advances
AAAS

Supplementary Materials for

Hedgehog-interacting protein orchestrates alveologenesis and protects against bronchopulmonary dysplasia and emphysema

Datian Ye *et al.*

Corresponding author: Chaoqun Wang, cqwang@simm.ac.cn

Sci. Adv. **11**, eadu2958 (2025)
DOI: 10.1126/sciadv.adu2958

The PDF file includes:

Figs. S1 to S6
Legends for tables S1 to S5

Other Supplementary Material for this manuscript includes the following:

Tables S1 to S5

fig. S1

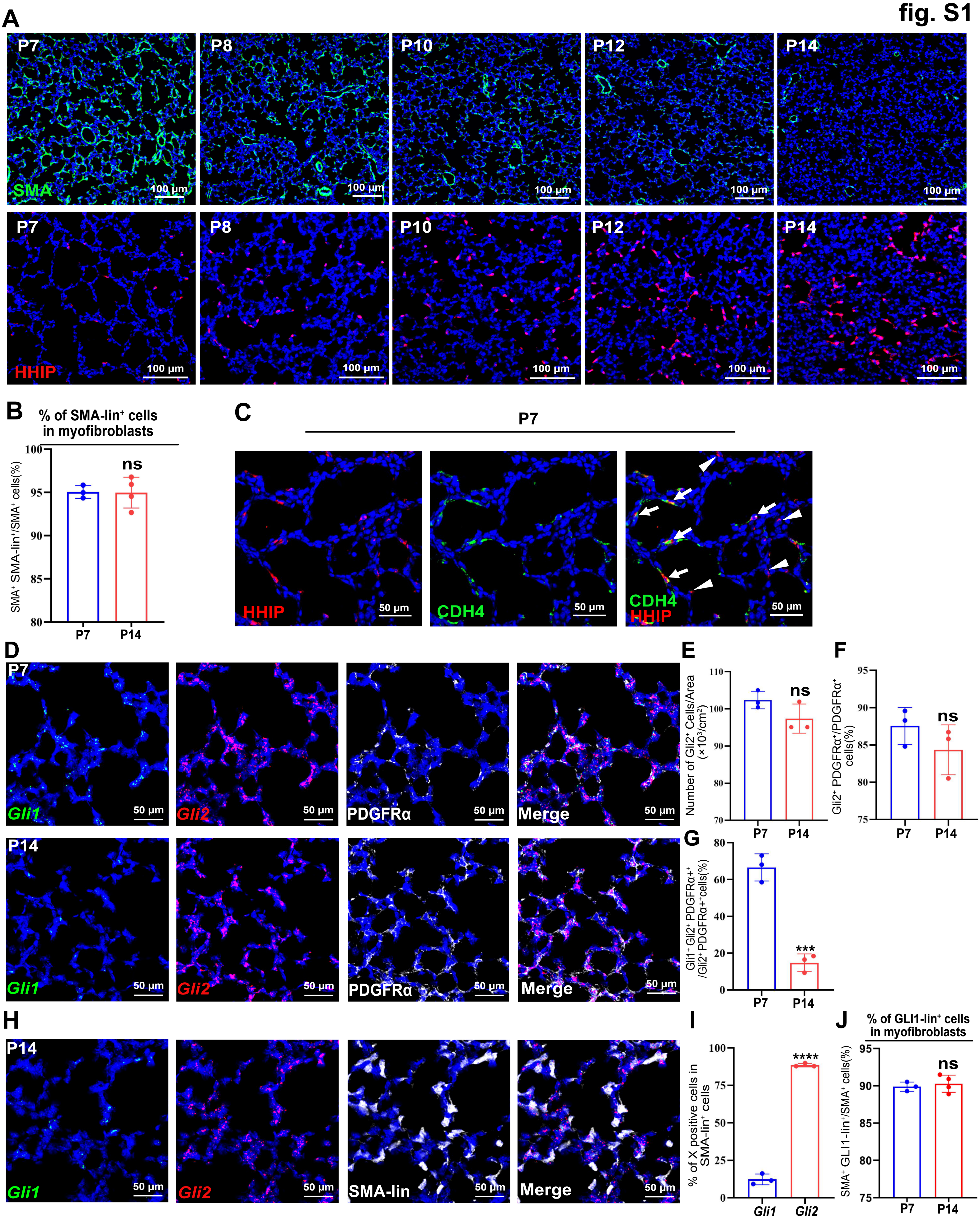
A
P7
P8
P10
P12
P14
SMA
HHIP
100 μm
B
% of SMA-lin+ cells in myofibroblasts
SMA+ SMA-lin+/SMA+ cells(%)
ns
P7
P14
C
P7
HHIP
CDH4
CDH4 HHIP
50 μm
D
Gli1
Gli2
PDGFRα
Merge
50 μm
E
Number of Gli2+ Cells/Area (×10³/cm²)
ns
F
Gli2+ PDGFRα+/PDGFRα+ cells(%)
ns
G
Gli1+ Gli2+ PDGFRα+ /Gli2+ PDGFRα+ cells(%)
***
H
SMA-lin
I
% of X positive cells in SMA-lin+ cells
****
J
% of GLI1-lin+ cells in myofibroblasts
SMA+ GLI1-lin+/SMA+ cells(%)
ns

fig. S1. Myofibroblast transition during alveologenesis.

(**A**) IF analysis of SMA and HHIP expression in the alveoli of postnatal mice at the indicated time points. (**B**) Histology quantification of the percentage of SMA-lineage cells in total myofibroblasts in the alveoli of P7 and P14 mice. (**C**) IF analysis of HHIP and CDH4 expression in the alveoli. Arrow: $HHIP^{+}$ $CDH4^{+}$ cells; Arrowhead: $HHIP^{+}$ $CDH4^{-}$ cells. (**D**) RNA *in situ* analysis of *Gli1* and *Gli2*, and IF analysis of PDGFRα in the alveoli. (**E**) Histology quantification of $Gli2^{+}$ cells in the alveoli of P7 and P14 mice. (**F**) Percentage of $Gli2^{+}$ $PDGFR\alpha^{+}$ in total $PDGFR\alpha^{+}$ cells. (**G**) Percentage of $Gli1^{+}$ $Gli2^{+}$ $PDGFR\alpha^{+}$ in total $Gli2^{+}$ $PDGFR\alpha^{+}$ cells. (**H**) Analysis of *Gli1* and *Gli2* expression (RNA *in situ*) and SMA-lin cells in the alveoli. (**I**) Percentage of $Gli1^{+}$ and $Gli2^{+}$ cells in total SMA-lin cells at P14. (**J**) Percentage of GLI1-lineage cells in total myofibroblasts in the alveoli of P7 and P14 mice. Each data point represents one mouse (**B**, **E** to **G, I,** and **J**) of an individual experiment. Data are expressed as Mean ± SD. ns, no significance; ***$p < 0.0005$; ****$p < 0.0001$.

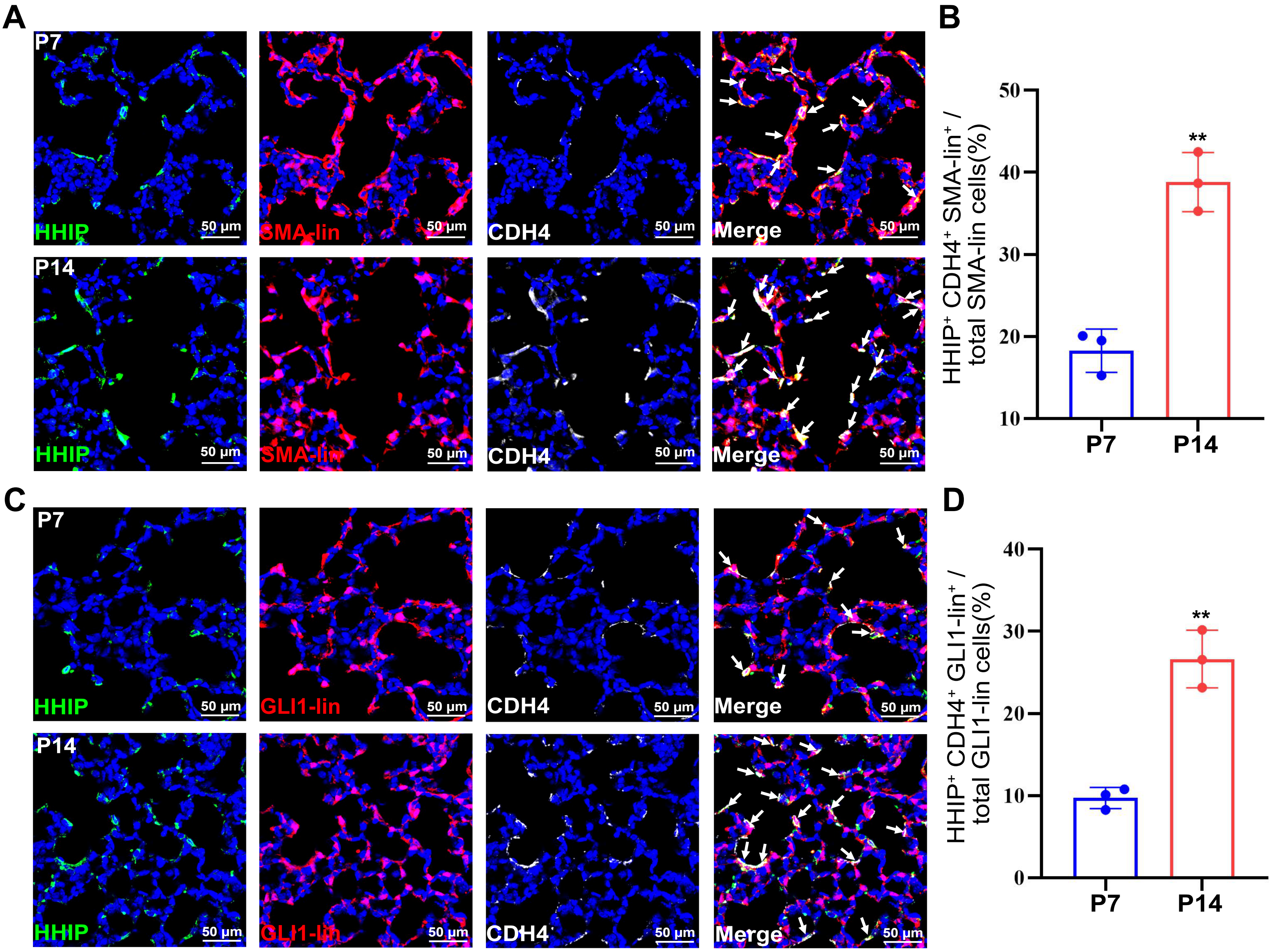

A
P7
HHIP
SMA-lin
CDH4
Merge
50 μm
P14
B
HHIP⁺ CDH4⁺ SMA-lin⁺ / total SMA-lin cells(%)
10
20
30
40
50
**
P7
P14
C
GLI1-lin
D
HHIP⁺ CDH4⁺ GLI1-lin⁺ / total GLI1-lin cells(%)
0

fig. S2. Ductal myofibroblasts in SMA-lin and GLI1-lin cells.

(**A**) IF analysis of HHIP and CDH4 expression, and SMA-lin cells in the alveoli of P7 and P14 mice. Arrow: SMA-lin$^+$ DMFs. (**B**) Percentage of HHIP$^+$ CDH4$^+$ cells in SMA-lin cells in the alveoli. (**C**) IF analysis of HHIP and CDH4 expression, and GLI1-lin cells in the alveoli of P7 and P14 mice. Arrow: Gli1-lin$^+$ DMFs. (**D**) Percentage of HHIP$^+$ CDH4$^+$ cells in GLI1-lin cells in the alveoli. Each data point represents one mouse (**B** and **D**) of an individual experiment. Data are expressed as Mean ± SD. $^{**}p < 0.005$.

fig. S3

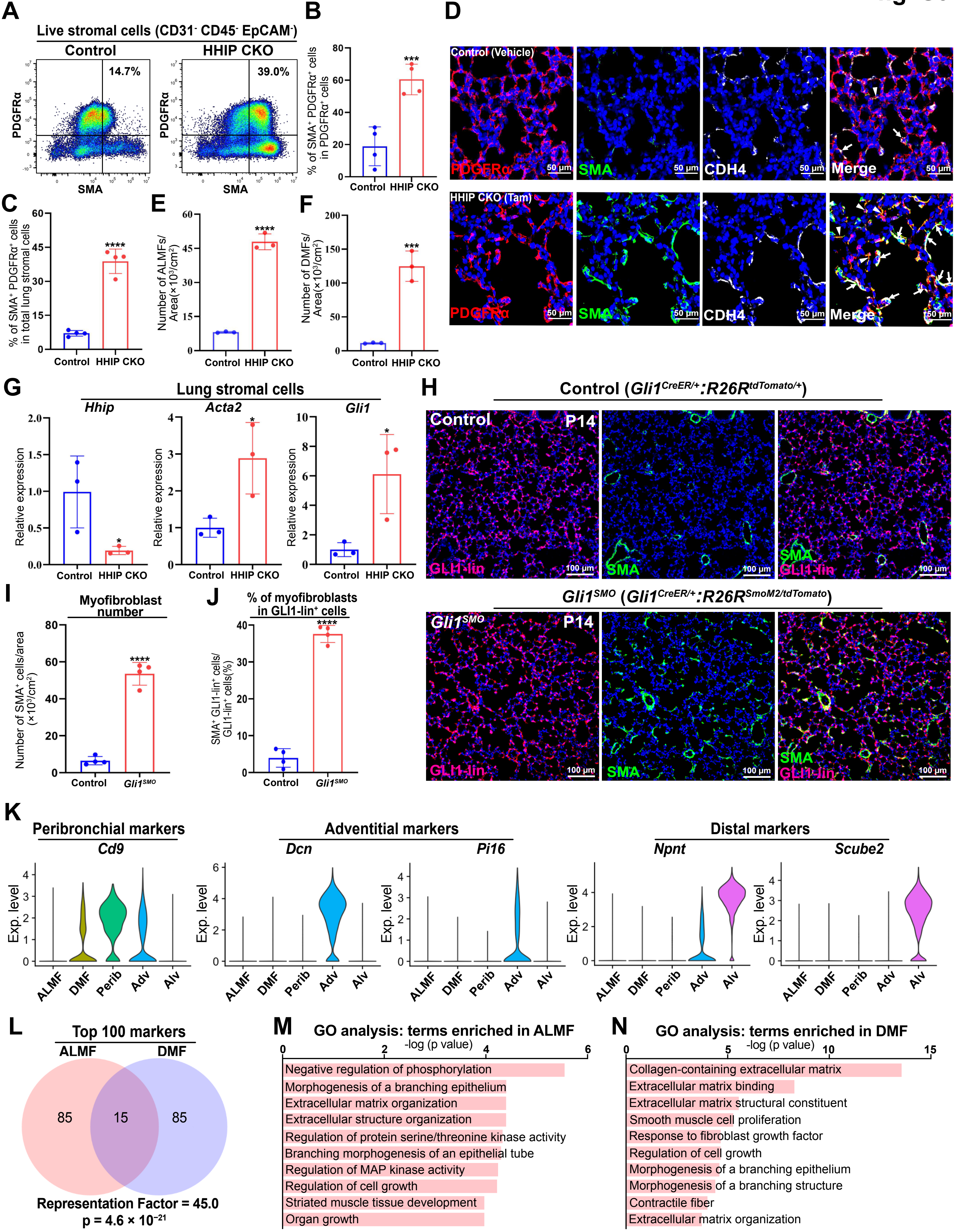
A
Live stromal cells (CD31- CD45- EpCAM-)
Control
HHIP CKO
14.7%
39.0%
PDGFRα
SMA
B
% of SMA+ PDGFRα+ cells in PDGFRα+ cells
***
Control
HHIP CKO
C
% of SMA+ PDGFRα+ cells in total lung stromal cells
****
E
Number of ALMFs/ Area(×10³/cm²)
F
Number of DMFs/ Area(×10³/cm²)
D
Control (Vehicle)
HHIP CKO (Tam)
PDGFRα
SMA
CDH4
Merge
50 μm
G
Lung stromal cells
Hhip
Acta2
Gli1
Relative expression
*
H
Control (Gli1CreER/+:R26RtdTomato/+)
Control
P14
GLI1-lin
SMA
100 μm
Gli1SMO (Gli1CreER/+:R26RSmoM2/tdTomato)
Gli1SMO
I
Myofibroblast number
Number of SMA+ cells/area (×10³/cm²)
J
% of myofibroblasts in GLI1-lin+ cells
SMA+ GLI1-lin+ cells/ GLI1-lin+ cells(%)
K
Peribronchial markers
Cd9
Adventitial markers
Dcn
Pi16
Distal markers
Npnt
Scube2
Exp. level
ALMF
DMF
Perib
Adv
Alv
L
Top 100 markers
ALMF
DMF
85
15
85
Representation Factor = 45.0
p = 4.6 × 10−21
M
GO analysis: terms enriched in ALMF
-log (p value)
Negative regulation of phosphorylation
Morphogenesis of a branching epithelium
Extracellular matrix organization
Extracellular structure organization
Regulation of protein serine/threonine kinase activity
Branching morphogenesis of an epithelial tube
Regulation of MAP kinase activity
Regulation of cell growth
Striated muscle tissue development
Organ growth
N
GO analysis: terms enriched in DMF
-log (p value)
Collagen-containing extracellular matrix
Extracellular matrix binding
Extracellular matrix structural constituent
Smooth muscle cell proliferation
Response to fibroblast growth factor
Regulation of cell growth
Morphogenesis of a branching epithelium
Morphogenesis of a branching structure
Contractile fiber
Extracellular matrix organization

fig. S3. Hh activation blocks myofibroblast transition.

(**A**) Flow cytometry analysis of SMA$^+$ cells in lung stromal cells of *Hhip*-deleted and control mice at P14. (**B**) Percentage of SMA$^+$ PDGFRα$^+$ in total PDGFRα$^+$ lung stromal cells, analyzed by flow cytometry. (**C**) Percentage of SMA$^+$ PDGFRα$^+$ in total lung stromal cells (live CD31$^-$ CD45$^-$ EpCAM$^-$), analyzed by flow cytometry. (**D**) IF analysis of SMA, PDGFRα, and CDH4 in the alveoli of *Hhip*-deleted and control mice at P14. Arrow: DMFs; Arrowhead: ALMFs. (**E and F**) Number of ALMFs (CDH4$^-$ SMA$^+$ PDGFRα$^+$, **E**) and DMFs (CDH4$^+$ SMA$^+$ PDGFRα$^+$, **F**) per unit alveolar area of *Hhip*-deleted and control mice at P14. (**G**) qPCR analysis of *Hhip*, *Acta2*, and *Gli1* expression in lung stromal cells isolated from *Hhip*-deleted and control mice. (**H**) IF analysis of SMA in Hh-activated (*Gli1^{SMO}*, *Gli1$^{CreER/+}$:R26R$^{SmoM2/tdT}$*) and control (*Gli1$^{CreER/+}$:R26R$^{tdT/+}$*) GLI1-lin cells. (**I**) Number of myofibroblasts in *Gli1^{SMO}* and control mice. (**J**) Percentage of myofibroblasts in total GLI1-lin cells. (**K**) Violin plots showing the expression of *Cd9*, *Dcn*, *Pi16*, *Npnt*, and *Scube2* in different lung stromal subsets. (**L**) Comparison of top 100 markers in ALMFs and DMFs with statistical comparison for degree of gene overlap of two independent gene sets denoted by representation factor (see methods). (**M** and **N**) Gene Ontology (GO) analysis of signature genes of ALMFs (**M**) and DMFs (**N**). Each data point represents one mouse (**B**, **C**, **E** to **G**, **I**, and **J**) of an individual experiment. Data are expressed as Mean ± SD. *$p < 0.05$; ***$p < 0.0005$; ****$p < 0.0001$.

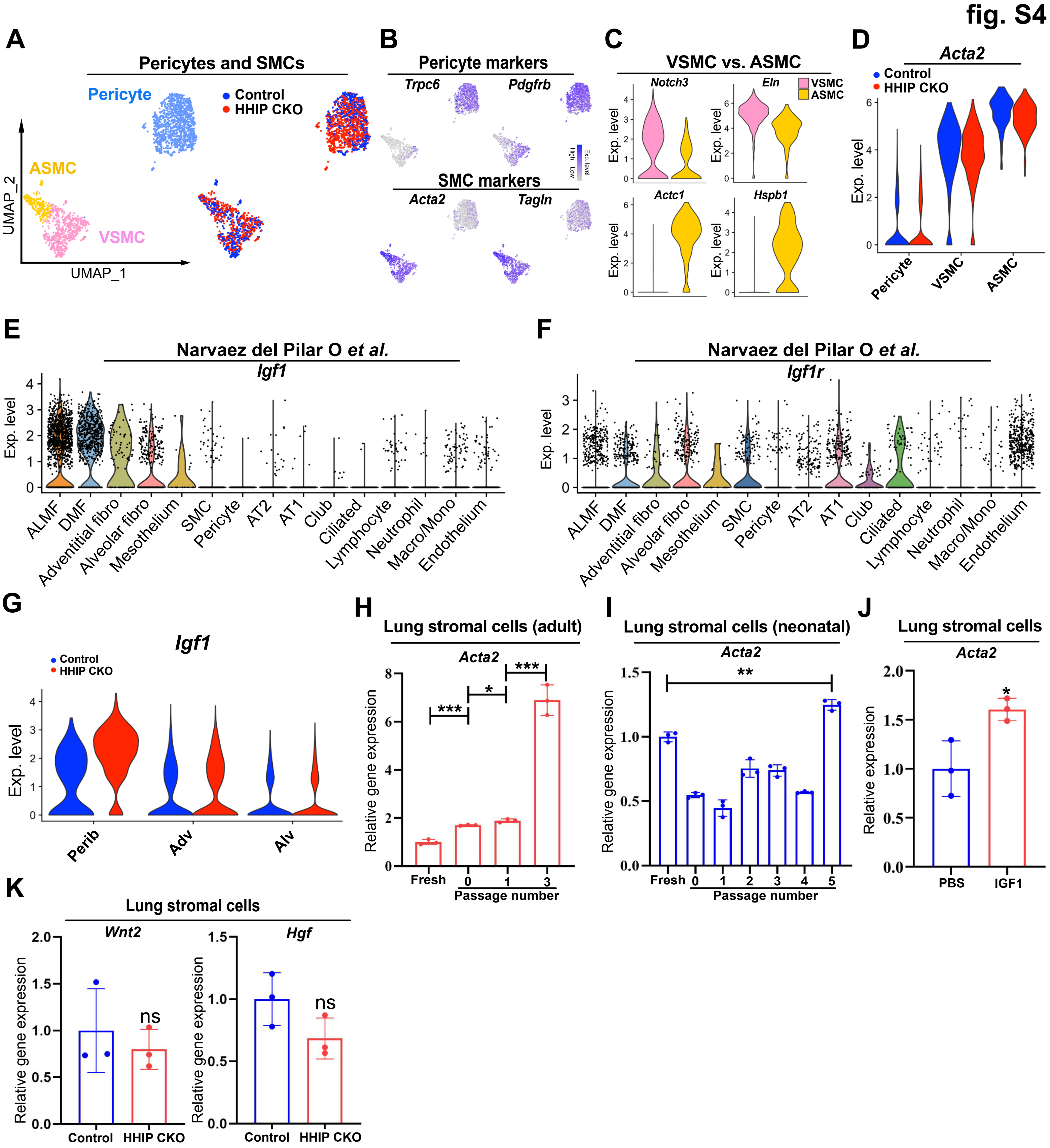
fig. S4
A
Pericytes and SMCs
Pericyte
Control
HHIP CKO
ASMC
VSMC
UMAP_1
UMAP_2
B
Pericyte markers
Trpc6
Pdgfrb
SMC markers
Acta2
Tagln
C
VSMC vs. ASMC
Notch3
Eln
VSMC
ASMC
Actc1
Hspb1
Exp. level
D
Acta2
Control
HHIP CKO
Exp. level
Pericyte
VSMC
ASMC
E
Narvaez del Pilar O et al.
Igf1
Exp. level
ALMF
DMF
Adventitial fibro
Alveolar fibro
Mesothelium
SMC
Pericyte
AT2
AT1
Club
Ciliated
Lymphocyte
Neutrophil
Macro/Mono
Endothelium
F
Narvaez del Pilar O et al.
Igf1r
G
Igf1
Control
HHIP CKO
Exp. level
Perib
Adv
Alv
H
Lung stromal cells (adult)
Acta2
Relative gene expression
Fresh
Passage number
I
Lung stromal cells (neonatal)
Acta2
Relative gene expression
Passage number
J
Lung stromal cells
Acta2
Relative expression
PBS
IGF1
K
Lung stromal cells
Wnt2
Hgf
Relative gene expression
ns
Control
HHIP CKO

fig. S4. scRNA-seq analysis of pericytes and smooth muscle cells of *Hhip*-deleted vs. control mice, along with *Igf1/Igf1r* expression in the lungs.

(**A** and **B**) UMAP showing the cell clusters in pericytes and smooth muscle cells (SMCs) of *Hhip*-deleted and control mice at P14. Airway smooth muscle cells (ASMCs); Vascular smooth muscle cells (VSMCs). (**C**) Violin plots showing the expression of *Notch3*, *Eln*, *Actc1*, and *Hspb1* in VSMCs vs. ASMCs). (**D**) Violin plots showing the expression of *Acta2* in pericytes, VSMCs, and ASMCs of *Hhip*-deleted and control mice at P14. (**E** and **F**) scRNA-seq analysis of *Igf1* (**E**) and *Igf1r* (**F**) expression in the lungs (P7 and P13). (**G**) *Igf1* expression in Perib, Adv, and Alv fibroblasts of *Hhip*-deleted and control mice at P14. (**H** and **I**) qPCR analysis of *Acta2* expression in fresh and passaged lung stromal cells of adult (**H**) and P12 neonatal (**I**) mice. (**J**) qPCR analysis of *Acta2* expression in lung stromal cells treated with PBS or IGF1 recombinant protein. (**K**) qPCR analysis of *Wnt2* and *Hgf* expression in lung stromal cells of *Hhip*-deleted and control mice at P14. All in vitro experiments have been repeated at least one time with consistent results for validation. Each data point represents one mouse (**K**) of an individual experiment. Data are expressed as Mean ± SD. ns, no significance; *$p < 0.05$; **$p < 0.005$; ***$p < 0.0005$.

fig. S5

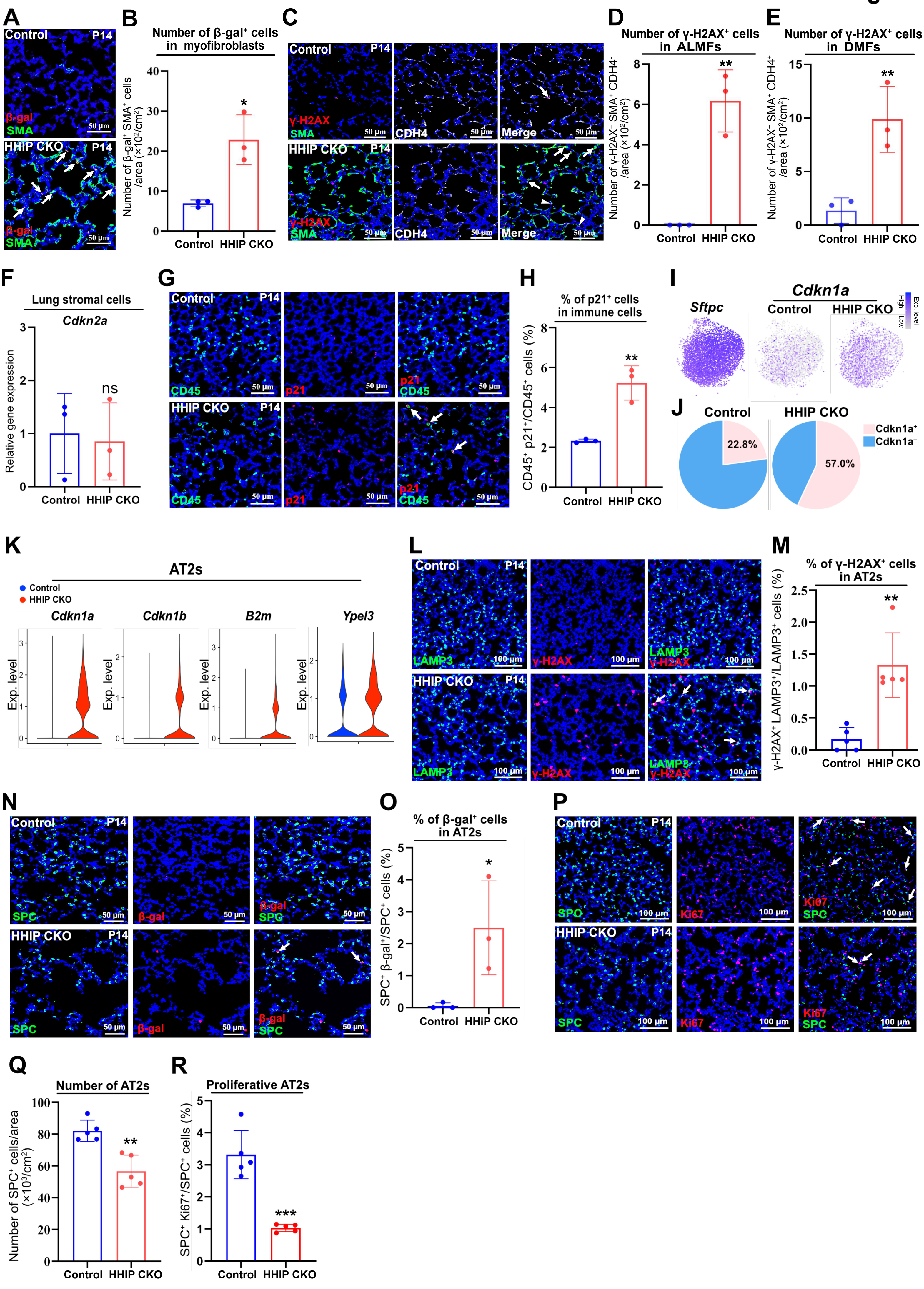
A
Control
P14
β-gal
SMA
50 μm
HHIP CKO
B
Number of β-gal⁺ cells in myofibroblasts
Number of β-gal⁺ SMA⁺ cells /area (×10²/cm²)
Control
HHIP CKO
C
γ-H2AX
CDH4
Merge
D
Number of γ-H2AX⁺ cells in ALMFs
Number of γ-H2AX⁺ SMA⁺ CDH4⁻ /area (×10²/cm²)
E
Number of γ-H2AX⁺ cells in DMFs
Number of γ-H2AX⁺ SMA⁺ CDH4⁺ /area (×10²/cm²)
F
Lung stromal cells
Cdkn2a
Relative gene expression
ns
G
CD45
p21
H
% of p21⁺ cells in immune cells
CD45⁺ p21⁺/CD45⁺ cells (%)
I
Sftpc
Cdkn1a
Exp. level
High
Low
J
22.8%
57.0%
Cdkn1a⁺
Cdkn1a⁻
K
AT2s
Cdkn1a
Cdkn1b
B2m
Ypel3
L
LAMP3
100 μm
M
% of γ-H2AX⁺ cells in AT2s
γ-H2AX⁺ LAMP3⁺/LAMP3⁺ cells (%)
N
SPC
O
% of β-gal⁺ cells in AT2s
SPC⁺ β-gal⁺/SPC⁺ cells (%)
P
Ki67
Q
Number of AT2s
Number of SPC⁺ cells/area (×10³/cm²)
R
Proliferative AT2s
SPC⁺ Ki67⁺/SPC⁺ cells (%)

fig. S5. Cell senescence induced by *Hhip*-deletion.

(**A**) IF analysis of SMA and senescence β-galactosidase staining in *Hhip*-deleted and control lungs at P14. Arrow: β-gal^+ SMA^+ cells. (**B**) Number of β-gal^+ SMA^+ cells per unit alveolar area of *Hhip*-deleted and control mice at P14. (**C**) IF analysis of γ-H2AX, SMA, and CDH4 in *Hhip*-deleted and control lungs. Arrow: γ-$H2AX^+$ DMFs; Arrowhead: γ-$H2AX^+$ ALMFs. (**D** and **E**) Number of γ-$H2AX^+$ ALMFs (**D**) and DMFs (**E**) per unit alveolar area of *Hhip*-deleted and control mice at P14. (**F**) qPCR analysis of *Cdkn2a* expression in lung stromal cells of *Hhip*-deleted and control mice at P14. (**G**) IF analysis of CD45 and p21 in *Hhip*-deleted and control lungs. Arrow: $CD45^+$ $p21^+$ cells. (**H**) Percentage of $p21^+$ cells in immune cells of *Hhip*-deleted and control lungs. (**I** and **J**) Feature plots and quantification of $Cdkn1a^+$ cells in AT2s of *Hhip*-deleted and control lungs. (**K**) Violin plots showing the expression of *Cdkn1a*, *Cdkn1b*, *Bem*, and *Ypel3* in AT2s of *Hhip*-deleted and control mice. (**L**) IF analysis of LAMP3 and γ-H2AX in *Hhip*-deleted and control lungs. Arrow: γ-$H2AX^+$ $LAMP3^+$cells. (**M**) Percentage of γ-$H2AX^+$ cells in AT2s. (**N**) IF analysis of SPC and senescence β-galactosidase staining in *Hhip*-deleted and control lungs. Arrow: β-gal^+ SPC^+ cells. (**O**) Percentage of β-gal^+ cells in AT2s. (**P**) IF analysis of SPC and Ki67 in *Hhip*-deleted and control lungs. Arrow: $Ki67^+$ SPC^+ cells. (**Q** and **R**) Quantification of the number of AT2s and percentage of proliferative AT2s in *Hhip*-deleted and control lungs. Each data point represents one mouse (**B**, **D**, **E**, **F**, **H**, **M**, **O**, **Q**, and **R**) of an individual experiment. Data are expressed as Mean ± SD. ns, no significance; *$p < 0.05$; **$p < 0.005$; ***$p < 0.0005$.

fig. S6

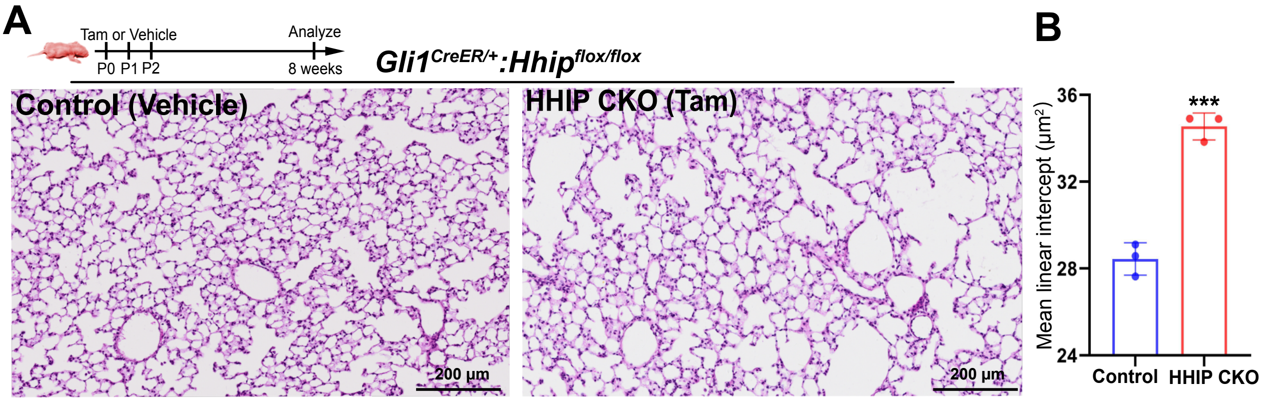
A
Tam or Vehicle
Analyze
P0 P1 P2
8 weeks
Gli1CreER/+:Hhipflox/flox
Control (Vehicle)
HHIP CKO (Tam)
200 µm
200 µm
B
Mean linear intercept (µm2)
36
32
28
24
***
Control
HHIP CKO

fig. S6. Neonatal *Hhip* deletion induces emphysema in adult mice.

(**A** and **B**) H&E images and MLI quantification of the lungs from adult *Gli1*^{HHIPCKO} and control mice. Each data point represents one mouse (**B**) of an individual experiment. Data are expressed as Mean ± SD. ***p < 0.0005.

table S1. Signature genes for different fibroblast subsets.

table S2. List of DEGs in ductal myofibroblasts of the *Hhip*-deleted lung vs. the control.

table S3. List of DEGs in alveolar myofibroblasts of the *Hhip*-deleted lung vs. the control.

table S4. List of DEGs in AT2s of the *Hhip*-deleted lung vs. the control.

table S5. List of qPCR primers.
